# Cancer nanotechnology: current status and perspectives

**DOI:** 10.1186/s40580-021-00282-7

**Published:** 2021-11-02

**Authors:** Jessica A. Kemp, Young Jik Kwon

**Affiliations:** 1 Department of Pharmaceutical Sciences, School of Pharmacy and Pharmaceutical Sciences, University of California, Irvine, CA 92697 USA; 2 Department of Chemical and Biomolecular Engineering, School of Engineering, University of California, Irvine, CA 92697 USA; 3 Department of Biomedical Engineering, School of Engineering, University of California, Irvine, CA 92697 USA; 4 Department of Molecular Biology and Biochemistry, School of Biological Sciences, University of California, Irvine, CA 92697 USA

**Keywords:** Cancer, Clinical translation, Diagnostics, Nanomedicine, Radiation, Theranostics

## Abstract

Modern medicine has been waging a war on cancer for nearly a century with no tangible end in sight. Cancer treatments have significantly progressed, but the need to increase specificity and decrease systemic toxicities remains. Early diagnosis holds a key to improving prognostic outlook and patient quality of life, and diagnostic tools are on the cusp of a technological revolution. Nanotechnology has steadily expanded into the reaches of cancer chemotherapy, radiotherapy, diagnostics, and imaging, demonstrating the capacity to augment each and advance patient care. Nanomaterials provide an abundance of versatility, functionality, and applications to engineer specifically targeted cancer medicine, accurate early-detection devices, robust imaging modalities, and enhanced radiotherapy adjuvants. This review provides insights into the current clinical and pre-clinical nanotechnological applications for cancer drug therapy, diagnostics, imaging, and radiation therapy.

## Introduction

Cancer devastates tens of millions of lives each year despite great advances in medicine and technology [1, 2]. Decades of research continuously reveal the ever-dynamic nature of the disease, and although treatment options have improved, severe side effects from harsh chemotherapies persist [3, 4]. Particularly, when aggressive cancers lie dormant then re-emerge, patients suffer when the need arises for more aggressive therapies [5–7]. One of the greatest challenges in finding a successful cancer treatment is the pervasive emergence of resistance mechanisms. Upon shutdown of initial oncogenic routes, resistance mechanisms are activated in parallel signaling pathways and re-route to allow for cancer to thrive [8, 9]. Heterogeneity can be found within different tumor cells, between patient tumors, amongst genetic mutations, and epigenetic patterns, all of which can limit responses to therapeutics, further allowing for drug resistance [10–13]. Clonal heterogeneity affects overall tumor biology and is known to drive metastasis and cancer progression [14]. Although new targets and therapies can advance cancer treatments, the dynamic nature of cancer finds a way to survive.

**Fig. 1:**
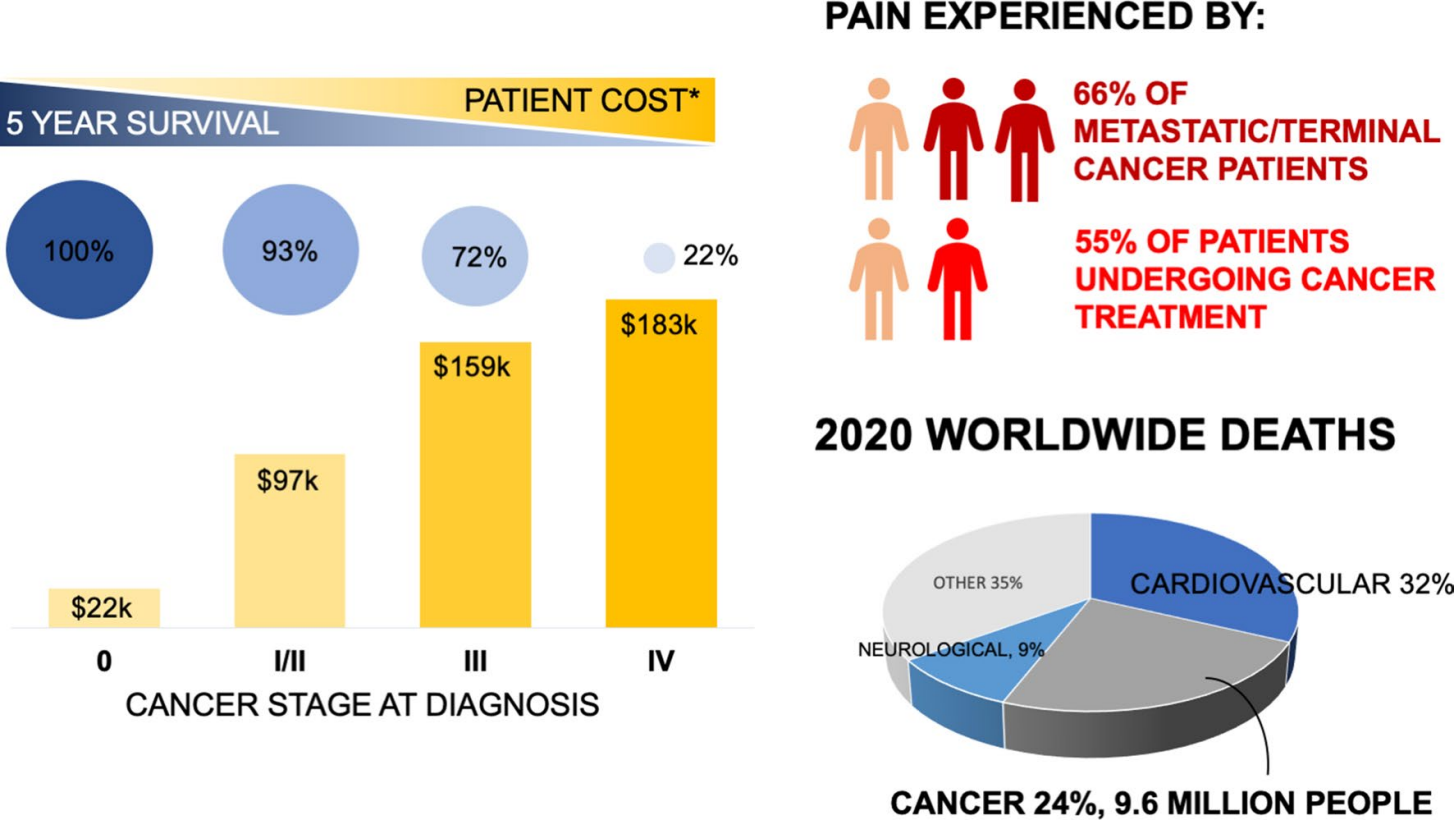
Late stage diagnoses for cancer results in significantly higher patient costs and decreased 5-year survival rates. The burden of cancer severely impacts patient quality of life with a majority experiencing pain directly from the disease and/or from treatment side effects. As the second leading cause of deaths worldwide, it is pertinent that new avenues are explored to improve cancer therapies and diagnostics

The strategy against cancer needs to shift from finding new therapies to improving existing therapies and diagnostics in innovative, effective, and plausible ways. Pain is experienced by 55% of patients undergoing cancer treatment and 66% of patients with advanced stage cancer [15]. Chemotherapies without distinct targeting mechanisms kill cancerous and noncancerous cells alike, therefore the systemic toxicity will continue to deteriorate patient quality of life [16, 17]. Furthermore, the benefits of early detection are clear. Cancer detected in early stages has a significantly higher 5-year survival rate, considerably lower overall cost to the patient, and typically less aggressive treatment course (Fig. 1) [18–20].

The solution may be found in nanotechnology: equipping existing therapies with better targeting capability, increasing localized drug efficacy, limiting systemic toxicity, improving diagnostic sensitivity, enhancing imaging, and refining radiation therapy [21–24]. Clinical translation of cancer nanomedicine dates back several decades, and the number of nano-based therapies and components for imaging, diagnostics, and radiation therapy in clinical use has steadily increased (Table 1) [25, 26]. For example, the CellSearch® system is the first FDA-approved diagnostic blood test which utilizes magnetic nanoparticles (NPs) targeting EpCAM and cell staining to identify circulating tumor cells [27]. Nano-based imaging contrast agents such as superparamagnetic iron oxide NPs (SPIONs) and Gadolinium (Gd)-based contrast agents enhance detection of tumor and imaging in vivo when using conventional scanning devices, such as magnetic resonance imaging (MRI), positron emission tomography (PET), and computed tomography (CT) [28].

**Table 1:** Nano-formulated cancer therapeutics currently on market(Adapted with permission [42])

Product name	Composition	Indications	First approval
Doxil/Caelyx	PEGylated liposomal doxorubicin	Myeloma, Kaposi’s sarcoma, breast, and ovarian cancer	US (1995)
DaunoXome	Liposomal daunorubicin	Kaposi’s sarcoma	US (1996)
Myocet	Liposomal doxorubicin	Breast cancer	Europe/Canada (2000)
Abraxane	Albumin-bound paclitaxel	Breast, non-small-cell lung, and pancreatic cancer	US (2005)
Lipusu	Liposomal paclitaxel	Breast and non-small-cell lung cancer	China (2006)
Nanoxel	Paclitaxel micellar	Solid tumors	India (2006)
Oncaspar	L-asparaginase conjugate	Acute lymphoblastic leukemia	US (2006)
DepoCyt	Liposomal cytarabine	Lymphoma, Leukemia	US (1999)
Genexol-PM	Paclitaxel micellar	Breast, non-small-cell lung, ovarian, and gastric cancer	South Korea (2007)
Mepact	Liposomal mifamurtide	Osteogenic sarcoma	Europe (2009)
NanoTherm	Iron oxide NPs	Brain tumors	Europe (2011)
Marqibo	Liposomal vincristine sulfate	Acute lymphoblastic leukemia	US (2012)
ONIVYDE	Liposomal irinotecan	Advanced pancreatic cancer	US (2015)
DHP107	Paclitaxel lipid NPs (oral administration)	Gastric cancer	South Korea (2016)
Vyxeos	Liposomal daunorubicin and cytarabine	High-risk acute myeloid leukemia	US (2017)
Apealea	Paclitaxel micellar	Ovarian, peritoneal, and fallopian tube cancer	Europe (2018)
Hensify	Hafnium oxide NPs	Locally-advanced soft tissue sarcoma	Europe (2019)

Nanoformulations can counter resistance mechanisms by targeting multiple components with dual-drug loading, increasing specificity with triggered release, and utilizing physical modalities to eradicate cancerous cells [29, 30]. Nanoscale carriers can cross a tumor endothelium and passively accumulate in tumors owing to the leaky blood vessels and poor lymphatic drainage [31]. Furthermore, nanomaterials have unique physico-chemical properties which are employed in highly sensitive diagnostic tests, allowing for early detection of cancer and better patient prognosis [32, 33]. Cancer diagnostics are steadily moving away from invasive, complicated procedures to the direction of highly sensitive point-of-care liquid biopsies, where nanomaterials have demonstrated high utility for biomarker detection [34–36]. Certain properties also enable vast improvement of imaging techniques used for surgical guidance and tumor surveillance, enabling highly specific surgical resection and enhanced treatment monitoring [37]. Nanomaterials can function as radiosensitizers, creating highly specific and uniform radiation dosing to tumors while sparing healthy tissue [38]. The versatility and functionality of nanomaterials provide a multitude of applications for cancer drug treatments, diagnostics, imaging, and radiotherapy. Early detection, decreased radiation dosage, and improved therapeutic specificity can help eliminate the systemic toxicities associated with traditional methods and improve prognosis and patient quality of life [39–41].

## Principles of nanotechnology

**Fig. 2:**
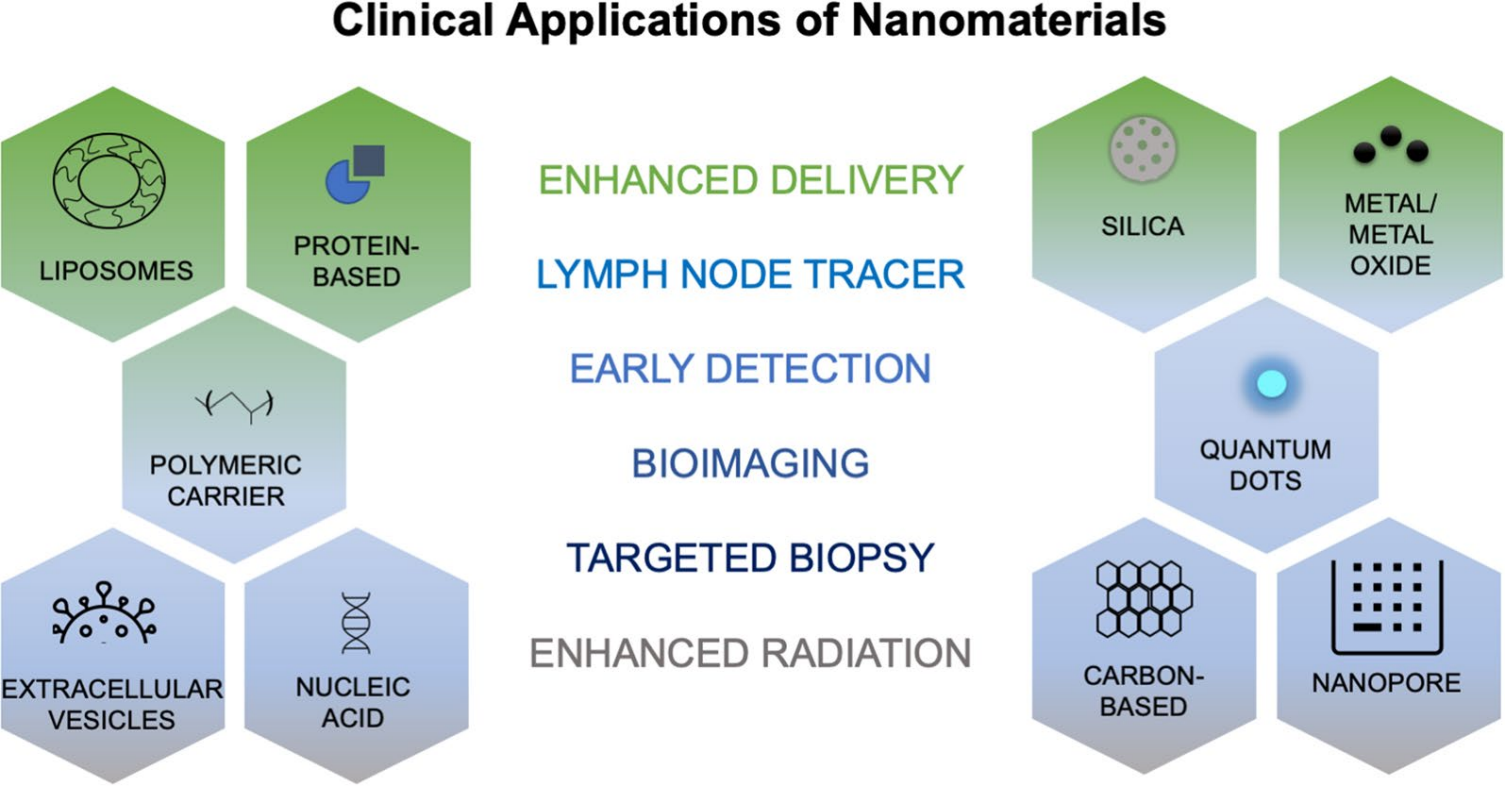
Examples of nanomaterials currently being investigated in clinical trials for various applications to improve therapeutic delivery, diagnostics, radiation therapy, and imaging modalities

Use of nanotechnology to improve therapeutics is no longer novel, in fact, there has been a steady increase in nanotechnology research as the benefits become more apparent [24, 26]. Currently approved cancer nanomedicines are predominantly liposomal formulations and drug conjugates (protein, polymer, and/or antibody) focused on improving pharmacokinetics and pharmacodynamics (PK/PD) of the free drug and utilizing passive targeting. There are many clinical studies currently investigating nanomaterials for therapeutic and diagnostic applications, including imaging modalities (Fig. 2) [43, 44]. Passive targeting for tumors is based upon the enhanced permeation and retention (EPR) effect, where NPs can preferentially accumulate within tumor vasculature [45]. Many tumors have leaky blood vessels with apertures suitable for NPs to pass through and accumulate within the tumor tissue [46]. However, the EPR effect is not the end-all solution: passive targeting does not eliminate drug action in healthy tissues nor the side effects that accompany systemic distribution [47]. There are physiological obstacles that prevent NPs from reaching their target, even without a diseased state, and can become even more complex to navigate for cancer patients [48]. Protein and lipid adsorption, blood flow rate, coronas, and phagocytic cells can reduce stability and delivery capability [49–52]. Interstitial pressure and extracellular matrices can also limit access to a tumor [53, 54]. Differences in cancer types can further complicate these issues, presenting a need to optimize formulation according to each kind [55]. First-generation nanomedicines have greatly improved pharmacokinetic (PK) profiles, solubility, bioavailability, and stability of major cancer therapeutics [56]. With the growing availability of technology and information, nanomaterials can broaden into new territory to incorporate highly specialized design and function. This enables the next generation of nanomedicine to incorporate combination therapies, specific targeting, triggered drug release, gene therapy, novel immunotherapy approaches, radiation, and multi-modal therapies. Furthermore, as scientific insights elucidate cancer initiation and survival mechanisms, nanotechnology will be a critical asset for improving diagnostics and bioimaging to halt metastasis.

Drugs can have vastly different biodistributive properties and relative concentrations, so it can be difficult to optimize dose coordination for combination therapies, and is further complicated by vast physiological variations in different types of cancer and amongst individual patients [44, 57]. Co-delivery of synergistic drugs within a single carrier can greatly improve synergistic potential as complementary action can occur in a coordinated fashion [30]. Nanomaterials such as lipid-based, polymeric, inorganic, carbon-based, biomacromolecular, and hydrogel can properly formulate multiple therapeutics with highly different chemical properties [58–61]. Multiple drugs may be engineered to be released simultaneously or in specific sequence depending on kinetics and mechanism of action, with drug release occuring through degradation of the carrier, drug desorption, diffusion through the nanoparticle matrix, or by triggered release [62, 63]. Specific targeting utilizes a nanocarrier or drug conjugate tethered with specific molecules that have high affinity for cancerous cells and lower affinity for healthy cells, lowering the likelihood of systemic toxicity [64, 65]. Antibody drug conjugates currently improve targeting, but targeted delivery of a nanocarrier may incorporate a higher dosage of drug and typically have more versatility for targeting modes using dynamic nanomaterials [66, 67]. For example, doxorubicin (DOX)-loaded immunoliposomes decorated with epidermal growth factor (EGF) to target EGF receptors (EGFR) are currently in clinical trials (ClinicalTrials.gov Identifier: NCT03603379). Specific targeting can also be utilized for tumor imaging, for example, probes targeting somatostatin receptors overexpressed in neuroendocrine tumors and activated only within the tumor microenvironment (TME) [68].

**Fig. 3:**
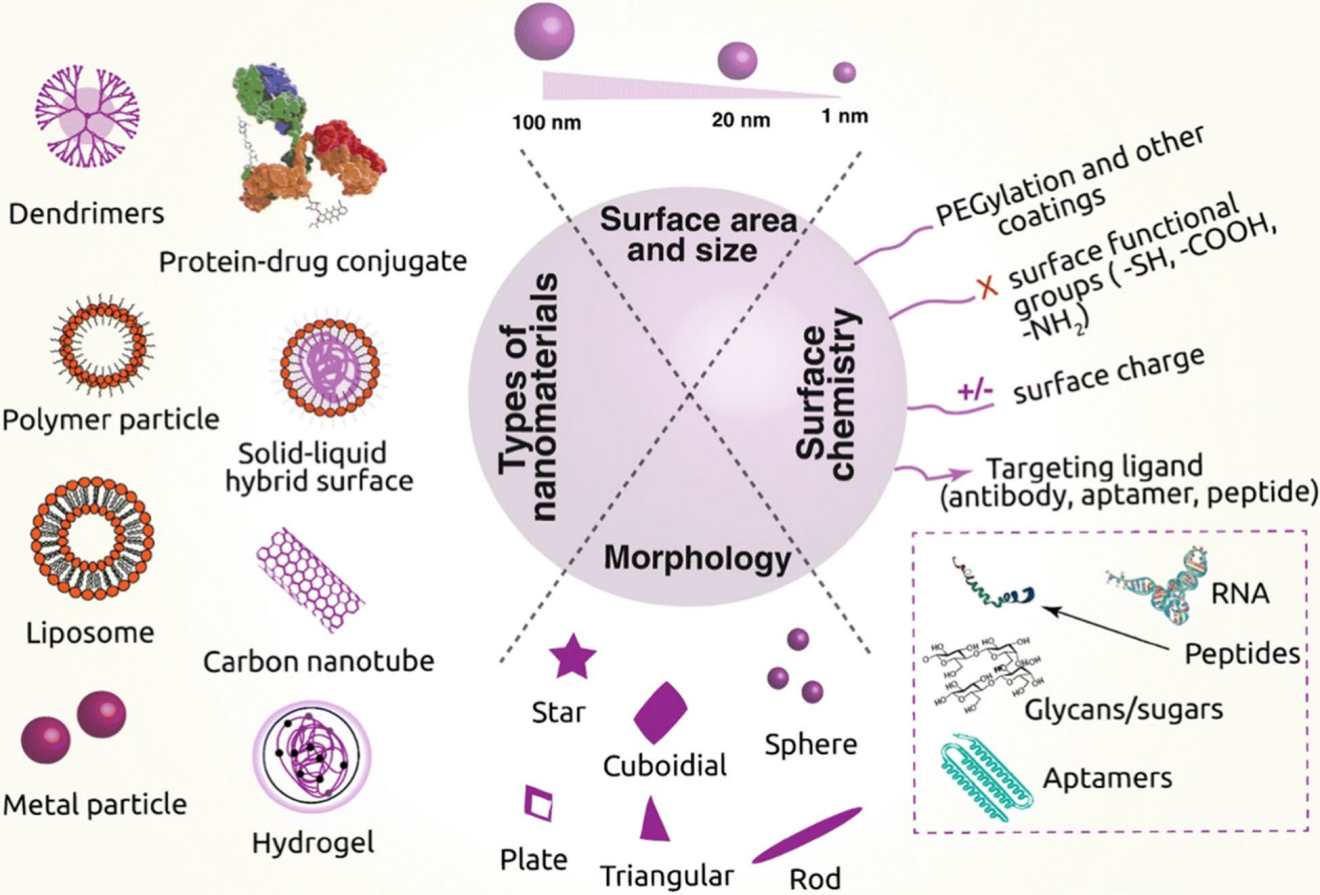
Schematic representation of versatile nanoformulations employed in cancer therapy and diagnostics, including specific physical formulation and surface chemistry for improved targeting. Reprinted with permission, [71] https://doi.org/10.1186/s40580-019-0193-2

Nanocarriers must be able to protect the cargo from degradation, achieve prolonged circulation, avoid the reticuloendothelial system uptake, and efficiently deliver to the target cells [69, 70]. Therefore, engineering of the nanoformulation requires proper selection of carrier materials, choice of ligand, and optimal density of ligand on the nanocarrier’s surface (Fig. 3) [71]. Certain therapies require intracellular delivery and while others utilize cellular membrane diffusion, so specific mechanism of action further plays a critical role in optimizing nanoformulation. Under particular circumstances, targeting constituents of the TME can be sufficient to see improved drug efficacy and specificity [72, 73]. In addition to specific targeting, nanotechnology can improve therapeutic specificity through stimuli-responsive activation. Release of drugs occurs under precise chemical, biological, or physical conditions found within tumor environment or cancerous cells to limit off-target effects [24, 26]. Nanocarriers may be designed to release drugs under specific pH, glucose levels, specific enzymes, oxidative/reductive conditions, and ion concentration, or by external stimulation such as radiation, electric and magnetic fields, and hyperthermia [32, 74–77]. These same modalities may be exploited for imaging and diagnostic purposes as well, such as utilizing magnetic particles for MRI tumor imaging or theranostic applications [78, 79]. pH-responsive peptide-based NPs were recently engineered to morph into fibrils within the TME where they exhibited strong fluorescent signals and enhanced photodynamic therapy [80].

**Fig. 4:**
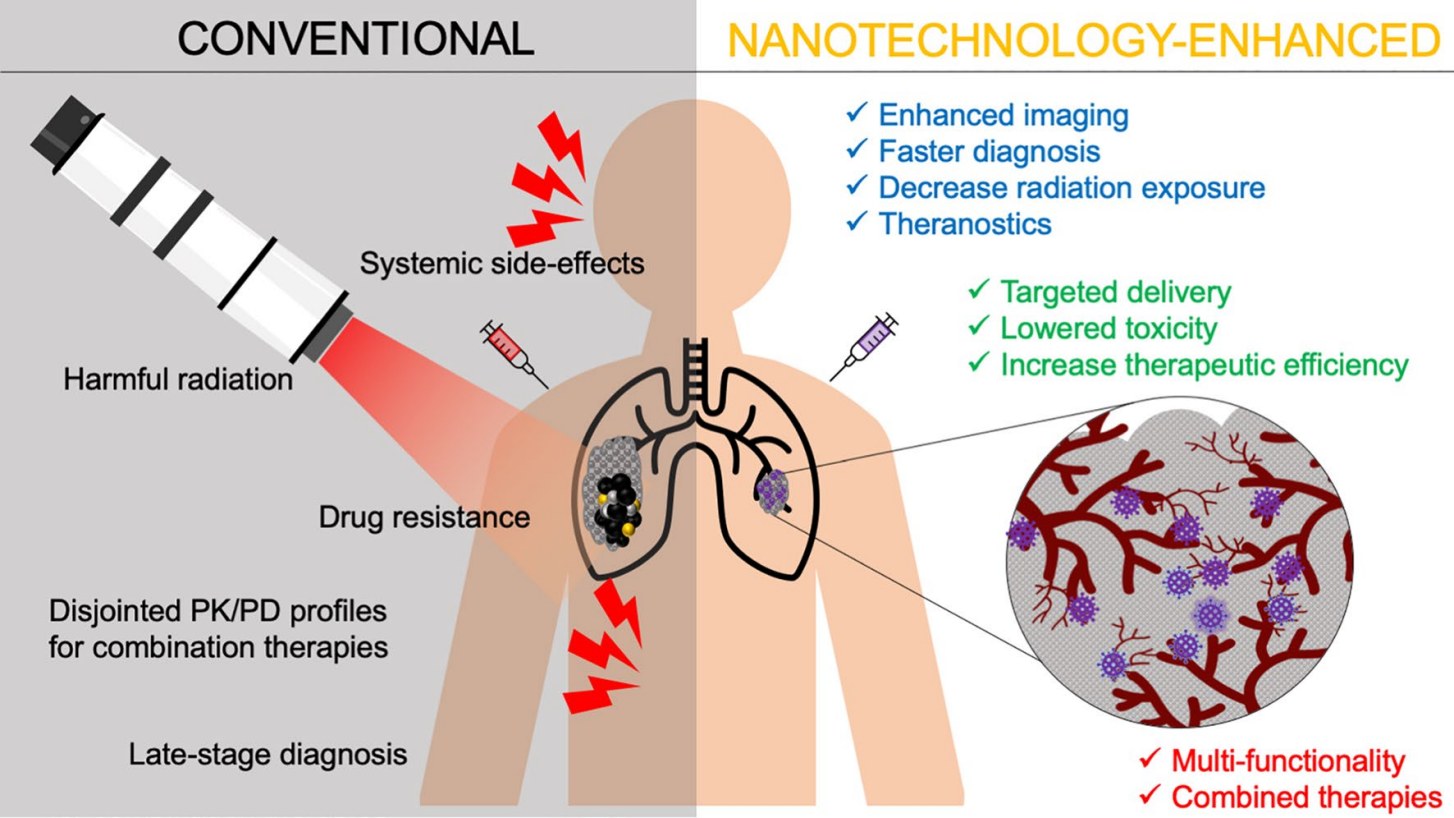
Nanotechnology provides many advantages over conventional anti-cancer drugs, radiation therapy, diagnostics, and imaging. Utilizing targeted delivery, nanomedicines can alleviate systemic toxicities while increasing therapeutic efficacy at the target site. Certain nanomaterials have intrinsic physico-chemical properties that enhance bioimaging, localize radiation therapy, facilitate early diagnoses, circumvent drug resistance, and enable multi-modal treatments

Intrinsic properties of certain nanomaterials are ideal for bioimaging, multi-modal therapies, and molecular detection for diagnostics [81, 82]. Fluorescent NPs have shown to be effective alternatives to traditional dyes, demonstrating high stability and decreased photobleaching [36]. Gd-based NPs have shown great utility as MRI and CT contrast agents and as radiosensitizers due to their paramagnetic property and high X-ray attenuation coefficient [83]. Gold NPs are ideal for creating highly selective, versatile, and sensitive biosensors, capable of optical and electrical detection, surface plasmon resonance, and fluorescence resonance energy transfer [84, 85]. Nanomaterials can enable early detection of circulating tumor cells (CTCs) from peripheral blood, as was shown using magnetic NPs functionalized with polyethyleneimine/protein corona or in a separate study, tannic acid [86, 87]. The vast range of nanotechnology applications can drastically improve cancer therapies and diagnostics, and this review provides an overview of current clinical applications and forthcoming technologies (Fig. 4).

## Applications of nanotechnology in cancer therapeutics

### Conventional cancer therapies

Chemotherapy remains the first-line treatment for most cancers, and drug discovery is constantly evolving and shifting toward cancer-specific targets [88]. Traditional chemotherapy drugs include alkylating agents and antibiotics to induce DNA damage, antimetabolites, mitotic inhibitors, and topoisomerase inhibitors to interfere with cellular replication [89]. Despite the high efficacy of traditional chemotherapies, patients suffer because of their non-specificity. Traditional chemotherapies are highly toxic to cancerous cells, but systemically affect healthy cells and induce harsh side effects for patients [90, 91].

There are specific signaling networks known to promote and sustain cancer, and a multitude of inhibitors currently exist and are under development to target enzymes within these pathways [92, 93]. Various inhibitors of tyrosine kinases, cyclin-dependent kinases, poly ADP-ribose polymerases, and proteasomes comprise the majority small-molecule drugs currently used in the clinic as targeted therapies [94]. Tumor growth and proliferation is fueled by components found in the TME such as immune and inflammatory cells, blood and lymphatic endothelial cells, cancer associated fibroblasts (CAFs), and bone marrow-derived mesenchymal stem cells [95–99]. Protein synthesis, glucose metabolism, and other key components of cell survival are often hyper-activated in the PI3K/Akt/mTOR pathways, often re-routing signals in response to initial therapies [100]. The RAS/RAF/MEK/ERK pathway initiates cell proliferation, differentiation, and development, thus multiple mutations are commonly found here across many cancer types [101, 102]. Mutation in RAS proteins is the one of the most commonly found in human cancers, and Sotorasib is the first KRAS targeting drug to receive FDA approval [103, 104]. Mutations in in EGFRs also contribute to oncogenesis, and there are approximately 14 EGFR-tyrosine kinase inhibitors (TKIs) on the market and/or in clinical trials [105, 106]. Targeting these pathways and factors responsible for cancer progression has become a focal point in developing new drug therapies, but new drug development costs billions of dollars and takes over a decade from development to FDA approval [107, 108].

Cytotoxic and targeted therapies can select for drug resistance, therefore making complete eradication nearly impossible [109]. Drug resistance may develop through alterations in drug metabolism, changes in efflux/influx, hyper-activated repair pathways, signal transduction re-routing, and mutated drug targets [110, 111]. Methods for overcoming drug resistance include multiple therapeutics, combination chemoradiotherapy, and personalized medicine [112]. Co-administration of drugs with different molecular targets can help modulate cancer cell mutations and possibly halt the cancer adaptation process [113]. Effective combinations have been found where a drug can heighten or re-introduce sensitivity of the cancer cells to an existing therapy, and new combinatorial treatments are consistently being investigated in clinical trials. However, limitations exist for combination treatments largely due to different PK/PD properties and disjointed uptake of the complementary drugs, which reduces their efficacy and synergistic action. Co-delivery of anti-cancer therapies within a single nanocarrier can alleviate these issues and increase the therapeutic index [56, 114]. In 2017, the U.S. Food and Drug Administration (FDA) approved VYXEOS, a liposomal formulation of cytarabine and daunorubicin at a fixed 5:1 molar ratio, for the treatment of adults with newly diagnosed acute myeloid leukemia (AML) with myelodysplasia-related changes and therapy-related AML [115]. The synergistic molar ratio of daunorubicin and cytarabine has been shown to enhance the killing of leukemia cells in vitro and in murine models. In preclinical studies, VYXEOS liposomes were preferentially taken up by leukemia cells than by normal bone marrow cells in a murine model [116]. Furthermore, the liposomes were strategically engineered to interact with receptors overexpressed in leukemic cells compared to the expression in normal bone marrow cells. This is a promising treatment option, but the need persists for more innovative technologies to combat drug resistance and therapy-related toxicities.

### Current clinical testing of nanoformulated therapeutics

**Fig. 5:**
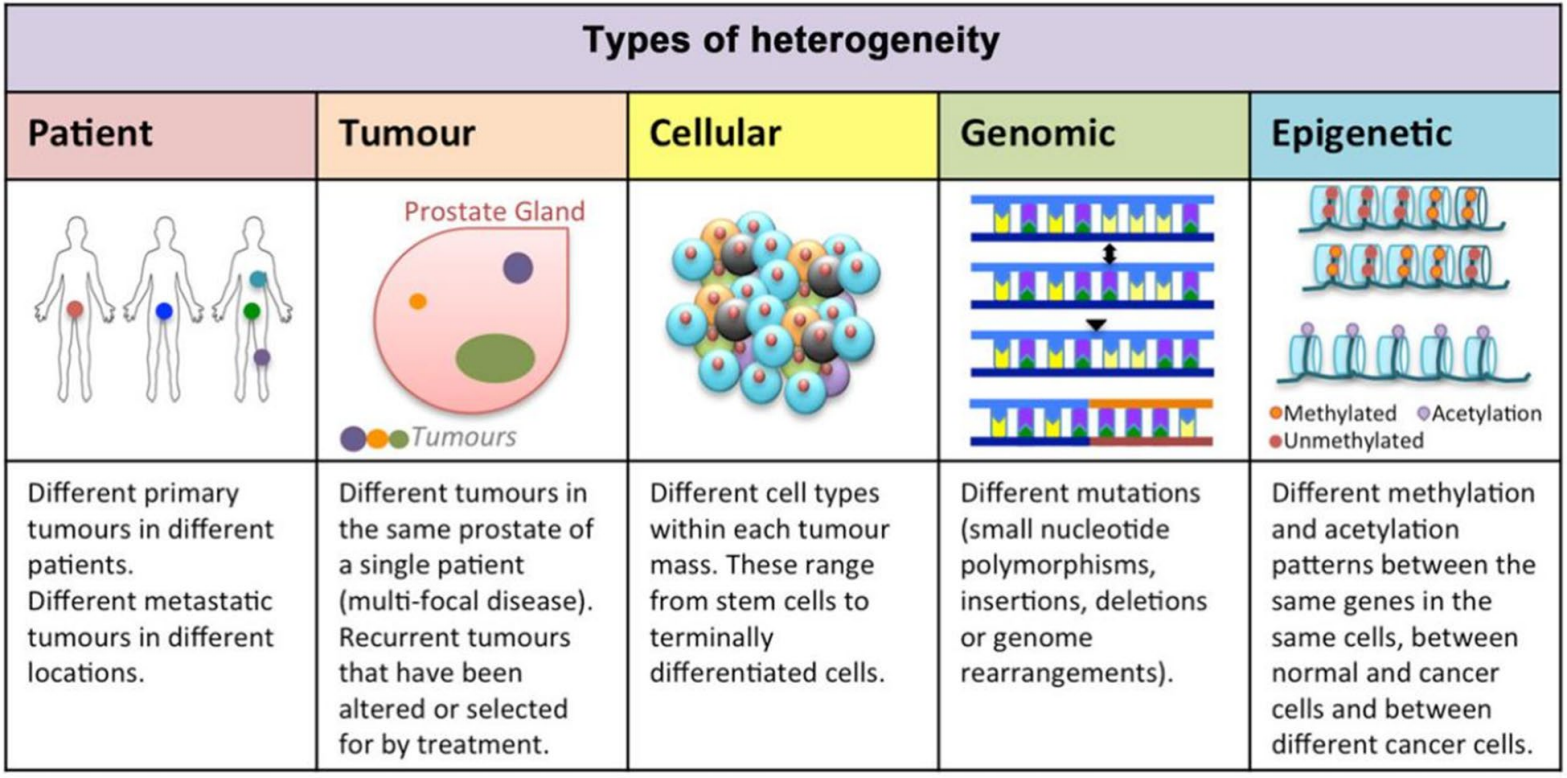
Multiple types of heterogeneity require consideration when treating cancer patients including patient tumors, multi-focal disease, intra-tumor cellular heterogeneity, genomic heterogeneity, and epigenetic heterogeneity. Reprinted with permission, [119] 10.20517/2394-4722.2017.34

Nanotechnology presents a unique set of tools to overcome both intrinsic and acquired drug resistance through various mechanisms and enabling the use of novel immunotherapies such as mRNA vaccines and specific targeting [117, 118]. Tumoral genetic diversity is accompanied by induced mutagenesis or differential sensitivity, and both can result in drug resistance and prolonged illness (Fig. 5) [119]. Various nanoformulations for cancer therapeutics are in clinical use including liposomes, polymer microspheres, protein conjugates, and polymer conjugates, and novel nanomaterials are being investigated for improved drug efficacy and targeting [118]. As aforementioned, targeted delivery is the pinnacle for cancer therapy since it can significantly lower toxicity associated with non-specific action. There are several new developments that incorporate targeting moieties which are currently being tested in clinical trials (Table 2).

**Table 2:** Current clinical studies investigating nanotechnology for cancer therapies

NCT Number	Title	Conditions	Interventions	Phases
NCT04852367	PanDox: Targeted Doxorubicin in pancreatic tumors	Pancreatic ductal adenocarcinoma/pancreatic cancer stage IV/pancreatic cancer non-resectable/pancreatic cancer metastatic	Device: focused ultrasound/Drug: Doxorubicin/Drug: ThermoDox	Phase 1
NCT04844983	A study to evaluate safety, efficacy of intralesional injection of STP705 in patients with isSCC	Squamous cell carcinoma in situ	Drug: STP705/Other: placebo saline	Phase 2
NCT04791228	A Pilot Study of Thermodox and MR-HIFU for Treatment of Relapsed Solid Tumors	Solid tumors/soft tissue sarcoma/ewing sarcoma/malignant epithelial neoplasm/rhabdomyosarcoma/wilms tumor/hepatic tumor/germ cell tumor/bone metastases	Device: magnetic resonance-guided high intensity focused ultrasound/Drug: Lyso-thermosensitive Liposomal Doxorubicin	Phase 2
NCT04751786	Dose escalation study of immunomodulatory NPs	Advanced solid tumor	Drug: PRECIOUS-01	Phase 1
NCT04676633	Open-label study for safety, tolerability, pk and anti-tumor activity of STP705 administered intratumorally in cholangiocarcinoma, hepatocellular carcinoma or liver metastases in subjects with advanced/metastatic or surgically unresectable solid tumors who are refractory to standard therapy	Hepatocellular carcinoma/liver metastases/cholangiocarcinoma	Drug: STP705	Phase 1
NCT04669808	Open label, dose escalation study for the safety and efficacy of STP705 in adult patients with basal cell carcinoma	Basal cell carcinoma	Drug: STP705	Phase 2
NCT04486833	TUSC2-NPs (GPX-001) and Osimertinib in patients with stage IV lung cancer who progressed on Osimertinib alone	Carcinoma, non-small-cell lung	Biological: Quaratusugene ozeplasmid—intravenous infusion/Drug: Osimertinib oral tablet	Phase 1/2
NCT04382898	PRO-MERIT (Prostate Cancer Messenger RNA Immunotherapy)	Prostate cancer	Biological: W_pro1/Drug: Cemiplimab	Phase 1/2
NCT04235101	Phase I study of SYD985 with Niraparib in patients with solid tumors	Solid tumor	Drug: SYD985 + Niraparib	Phase 1
NCT04205630	SYD985 in patients with HER2-expressing recurrent, advanced or metastatic endometrial carcinoma	Endometrial cancer	Drug: SYD985	Phase 2
NCT04202705	A first-in-human dose-escalation and expansion study with the antibody–drug conjugate SYD1875	Solid tumor	Drug: SYD1875	Phase 1
NCT04163094	Ovarian cancer treatment with a liposome formulated mRNA vaccine in combination with (neo-)adjuvant chemotherapy	Ovarian cancer	Drug: W_ova1 Vaccine	Phase 1
NCT04078295	A study of E7389 liposomal formulation (E7389-LF) plus Nivolumab in participants with solid tumor	Solid neoplasms	Drug: E7389-LF/Drug: Nivolumab	Phase 1/2
NCT03774680	Targeted polymeric NPs loaded with Cetuximab and decorated with Somatostatin analogue to colon cancer	Colon cancer/colo-rectal cancer	Drug: Cetuximab NPs/Drug: Oral approved anticancer drug	Phase 1
NCT03608631	iExosomes in treating participants with metastatic pancreas cancer with KrasG12D mutation	KRAS NP_004976.2:p.G12D/metastatic pancreatic adenocarcinoma/pancreatic ductal adenocarcinoma/stage IV pancreatic cancer AJCC v8	Drug: Mesenchymal stromal cells-derived exosomes with KRAS G12D siRNA	Phase 1
NCT03418480	HPV Anti-CD40 RNA vaccine	Carcinoma, squamous cell/head and neck neoplasm/cervical neoplasm/penile neoplasms malignant	Drug: HPV vaccine	Phase 1/2
NCT03313778	Safety, tolerability, and immunogenicity of mRNA-4157 alone in participants with resected solid tumors and in combination with Pembrolizumab in participants with unresectable solid tumors	Solid tumors	Biological: mRNA-4157/Biological: Pembrolizumab	Phase 1
NCT03262935	SYD985 vs. physician's choice in participants with HER2-positive locally advanced or metastatic breast cancer	Metastatic breast cancer	Drug: (vic-)trastuzumab duocarmazine/Drug: Physician's choice	Phase 3
NCT02769962	Trial of EP0057, a nanoparticle Camptothecin with Olaparib in people with relapsed/refractory small cell lung cancer	Urothelial carcinoma/urothelial cancer/lung neoplasms/small cell lung cancer/prostate cancer	Drug: EP0057/Drug: olaparib	Phase 1/2
NCT02389985	A Study of CRLX101(NLG207) in combination with weekly Paclitaxel in patients with recurrent or persistent epithelial ovarian, fallopian tube or primary peritoneal cancer	Ovarian cancer	Drug: CRLX101/Drug: Paclitaxel	Phase 1/2
NCT02316457	RNA-Immunotherapy of IVAC_W_bre1_uID and IVAC_M_uID	Breast Cancer (Triple Negative Breast Cancer (TNBC))	Biological: IVAC_W_bre1_uID/Biological: IVAC_W_bre1_uID/IVAC_M_uID	Phase 1
NCT02181075	Targeted chemotherapy using focused ultrasound for liver tumors	Liver tumor	Drug: ThermoDox¬Æ (LTLD)/Device: focused ultrasound of target liver tumor/Diagnostic Test: Pre-LTLD biopsy of target liver tumor/Diagnostic Test: Post-LTLD biopsy of target liver tumor/Diagnostic Test: Post-LTLD + FUS (Post-FUS) biopsy of target liver tumor/Device: thermometry of target tumor	Phase 1
NCT02112656	Study of ThermoDox with standardized radiofrequency ablation (RFA) for treatment of hepatocellular carcinoma (HCC)	Hepatocellular carcinoma	Drug: ThermoDox/Drug: Dummy infusion	Phase 3
NCT02022644	Study of convection-enhanced, image-assisted delivery of liposomal-irinotecan in recurrent high grade glioma	High grade glioma	Drug: Nanoliposomal irinotecan	Phase 1
NCT01861496	Phase I/II study to evaluate the safety and tolerability of LiPlaCis in patients with advanced or refractory tumors	Phase 1: advanced or refractory solid tumors/Phase 2 part: metastatic breast cancer, prostate cancer and skin cancer	Drug: LiPlaCis	Phase 1/2
NCT01591356	EphA2 siRNA in treating patients with advanced or recurrent solid tumors	Advanced malignant solid neoplasm	Drug: EphA2-targeting DOPC-encapsulated siRNA/Other: laboratory biomarker analysis/other: pharmacological study	Phase 1
NCT01437007	TKM 080,301 for primary or secondary liver cancer	Colorectal cancer with hepatic metastases/pancreas cancer with hepatic metastase/gastric cancer with hepatic metastase/breast cancer with hepatic metastase/ovarian cancer with hepatic metastase	Drug: TKM-080301	Phase 1
NCT01042379	I-SPY TRIAL: neoadjuvant and personalized adaptive novel agents to treat breast cancer	Breast neoplasms/breast cancer/breast tumors/angiosarcoma	Drug: Standard Therapy/Drug: AMG 386 with or without Trastuzumab/Drug: AMG 479 (Ganitumab) plus Metformin/Drug: MK-2206 with or without Trastuzumab/Drug: AMG 386 and Trastuzumab/Drug: T-DM1 and Pertuzumab/Drug: Pertuzumab and Trastuzumab/Drug: Ganetespib/Drug: ABT-888/Drug: Neratinib/Drug: PLX3397/Drug: Pembrolizumab—4 cycle/Drug: Talazoparib plus Irinotecan/Drug: Patritumab and Trastuzumab/Drug: Pembrolizumab—8 cycle/Drug: SGN-LIV1A/Drug: Durvalumab plus Olaparib/Drug: SD-101 + Pembrolizumab/Drug: Tucatinib plus trastuzumab and pertuzumab/Drug: Cemiplimab/Drug: Cemiplimab plus REGN3767/Drug: Trilaciclib with or without trastuzumab + pertuzumab/Drug: SYD985 ([vic-]trastuzumab duocarmazine)/Drug: Oral Paclitaxel + Encequidar + Dostarlimab (TSR-042) + Carboplatin with or without trastuzumab/Drug: Oral Paclitaxel + Encequidar + Dostarlimab (TSR-042) with or without trastuzumab	Phase 2
NCT00504998	Safety/efficacy study of Rexin-G to treat pancreatic cancer	Pancreatic cancer	Genetic: Rexin-G	Phase 1/2

#### Formulations for enhanced PK and specific targeting

Liposomes are a particularly advantageous class of nanomaterial for drug delivery applications, because of their ease of fabrication and drug loading, capacity for surface modification, and biocompatible components [44, 120, 121]. Liposomes are vesicles consisting of a lipid bilayer primarily composed of amphipathic phospholipids that encompass an aqueous interior. Properties of the liposome can be tuned depending on the phospholipid polar headgroup, length and hydrophobicity of the fatty acid tails, additional components in the membrane or on the surface, and type of synthetic or natural lipid [122]. Owing to the versatility and relative ease of manufacturing, liposomes are one of the most investigated nanomedicines for the treatment of many diseases. Doxil, a liposomal formulation of the highly toxic chemotherapy DOX, was the first of its kind approved by the FDA in 1995. One year later, another liposomal formulation of daunorubicin was approved, DaunoXome®, to treat advanced HIV-associated Kaposi sarcoma [44]. Marqibo®, a sphingomyelin/cholesterol liposomal formulation of vincristine sulfate, FDA approved in 2012, demonstrated enhanced PK/PD properties over vincristine as well as enhanced concentration in solid tumors. Depocyt® (Cytarabine/Ara-C), Myocet®(DOX), Mepact® (Mifamurtide), and Onivyde® (Irinotecan) are also liposomal therapeutics clinically approved for cancer treatment, making a total of only 7 currently on the market today. It should be noted, however, that Depocyt was on microscale, and has been discontinued in its use.

Cisplatin is one of the most widely used chemotherapies due to its efficacy against multiple cancer types but has severe side effects, demonstrating the critical need for specificity and re-formulation [123]. LiPlaCis is the first liposomal formulation with a triggered release mechanism to undergo clinical development in oncology, where selective hydrolysis occurs by tumor-expressed phospholipase A2–IIA isoenzyme, highly expressed in a multitude of human solid tumors including prostatic, pancreatic, colorectal, gastric, and breast cancers [124, 125]. LiPlaCis has an enhanced therapeutic window compared to cisplatin, with superior PK properties, greater potency, and an increased maximum tolerated dose (ClinicalTrials.gov Identifier: NCT01861496). Drug Response Prediction (DRP®) is used to significantly increase the probability of success in clinical trials. Patients undergo genetic screening of tumors, then are selected for the trial based upon those most likely to respond to treatment, providing a highly-defined patient group and subsequently lowering costs and risks [126]. DRP® has provided statistically significant prediction of drug treatment clinical outcome for cancer patients in 29 out of 37 clinical studies that were examined.

High grade gliomas are the most common brain tumor in adults, and median survival of 9–12 months for patients with newly diagnosed glioblastoma multiforme and 24–36 months for patients with anaplastic astrocytoma [127–129]. The currently available treatments for malignant gliomas are limited by low activity, drug resistance, brain damage from therapeutic modalities, and limited access to privileged intracranial sites [130]. A current Phase 1 clinical study is underway to investigate liposomal irinotecan and Gd administered with convection enhanced delivery (CED) (ClinicalTrials.gov Identifier: NCT02022644). Liposomal formulation enables for delivery across the blood brain barrier, and the Gd will provide capability for real-time delivery imaging. CED improves chemotherapeutic delivery to brain tumors intraparenchymally by utilizing fluid convection [131]. Through the maintenance of a pressure gradient from the delivery cannula tip to the surrounding tissues, CED is able to distribute small and large molecules, including liposomes, to clinically significant target volumes [131].

E7389-LF is a liposomal formulation of eribulin, a halichondrin-class microtubule dynamics inhibitor approved for treatment of advanced/metastatic breast cancer, and previously treated, unresected liposarcoma (ClinicalTrials.gov Identifier: NCT04078295) [132]. This nanomedicine is currently undergoing a Phase 1/2 clinical trial to evaluate safety and tolerability and to determine recommended Phase 2 dose of E7389-LF in combination with nivolumab in Phase 1b part, and to evaluate objective response rate of E7389-LF and nivolumab using RP2D in Phase 2 part in each tumor type. ThermoDox is a heat-activated lysolipid formulation of DOX designed to release the drug when heated to 40–45 °C [133]. It is currently in multiple clinical trials, including a completed Phase 3 trial, after initial trials showed a 2.1-year improvement in overall survival in liver cancer patients with single lesion (ClinicalTrials.gov Identifiers: NCT02181075, NCT04852367, NCT02112656, NCT04791228). Compared to I.V. administration of DOX, ThermoDox delivers up to 25 × more therapeutic to tumors and 5 × more than Doxil, the standard liposomal formulation of DOX. The majority of clinical trials currently investigating liposomal drugs for cancer therapy involve combination treatments with Doxil or DaunoXome, but not within a single carrier. Several clinical studies underway are utilizing liposomes for nucleic acid delivery, discussed in further detail in Sects. 3.2.2 and 3.2.3.

Paclitaxel (PTX), a naturally derived compound used against many types of cancer, has a unique mechanism to block cell cycle progression, prevent mitosis, and subsequently inhibit the growth of cancer cells. However, neuropathy, cardiotoxicity, and hepatotoxicity are potential side effects of PTX, posing a major downside to using the highly effective drug. Particularly, peripheral neuropathy includes shooting/burning pain (especially in hands and feet), sensory loss, numbness, and tingling [134]. Several nanoformulations have been developed to decrease the adverse effects of PTX and improve aqueous solubility without the use of Cremophor® EL, a common excipient in PTX formulation in solution, also known to cause toxicity [135]. Genexol-PM® is a PEG-b-poly(D,L-lactic acid) (PEG-PLA) micellar formulation of PTX approved in South Korea in 2007 for the treatment of breast cancer and NSCLC. It has shown lower toxicity than Taxol (PTX formulated with Cremophor® EL), with its maximum tolerated dose identified as 2 to 3 times that of Taxol. Nanoxel® is a polymeric amphiphilic micelle formulation approved for clinical use in India in 2006 and is currently undergoing clinical trials for FDA approval (ClinicalTrials.gov Identifier: NCT04066335), and Apealea, approved for use in the European Union in 2018, utilizes poly‐(l‐glutamic acid) conjugated to PTX. Unfortunately, peripheral neuropathy remains a clinical challenge despite improved formulations, further necessitating continued optimization [136].

Two major advantages of polymeric micelles are a desirable sub-50 nm hydrodynamic size and their relative ease of large-scale manufacturing. However, despite the utility of polymeric micelles, there are limitations in stability and drug retention once administered into the bloodstream [137]. Partial micellar dissolution occurs after micelles drop below the critical micelle forming concentration (CMC) in the blood, and certain blood components such as albumin and apolipoproteins can also initiate micelle dissociation and premature drug loss [138]. Formulations can be optimized to enhance stability and drug retention using strategies such as covalent core and/or shell crosslinking, drug conjugation via reversible bonds, zwitterionic polymer micelles, unimolecular micelle formulation, hydrogen-bond core complexation, and macrocylclic complexation [139]. But as with all drug formulations, with further complexity comes greater manufacturing and scale-up considerations. Hence, there are limited clinical trials on novel micellar formulations. A clinical trial (ClinicalTrials.gov Identifier: NCT01644890) for poly(ethylene glycol)-block-poly(aspartic acid) (PEG-b-pAsp) loaded PTX micelles (NK105) was recently completed where incidence of peripheral sensory neuropathy (PSN) was 1.4% vs. 7.5% (≥ Grade 3) for NK105 and PTX, respectively. NC-6300 is a micellar formulation of DOX, which is covalently conjugated to the carboxylic acid groups of PEG-b-p(b-Asp) via a a hydrazone bond, to enable drug release upon pH stimuli (pH < 5), and is undergoing a Phase 2 clinical investigation (ClinicalTrials.gov Identifier: NCT03168061). A poly(ethylene glycol)-block-poly(glutamic acid) (PEG-b-pGlu) micelle containing cisplatin (NC-6004) has undergone multiple clinical trials, and currently being evaluated for combination with Pembrolizumab for head and neck cancer patients who have failed platinum regimen (ClinicalTrials.gov Identifier: NCT03771820).

Polymer drug conjugates have been utilized in the pharmaceutical industry for decades, most notably, polyethylene glycol (PEG), for improving PK profiles by reducing immunogenicity, preventing degradation, and reducing plasma clearance [140]. There are a multitude of polymer-drug formulations, particularly as technological advances are made regarding design and synthetic procedures. Natural polymers such as chitosan, polysaccharides, polysialic acid, hyaluronic acid, and polypeptides have the advantage of greater biodegradability and biocompatibility over PEG [141]. Opaxio®, (formerly Xyotax®) utilizing a polyglutamate-PTX conjugate, was granted orphan drug designation by the FDA, and is currently in a Phase 3 clinical trial for treatment of patients with stage III/IV ovarian epithelial, peritoneal, or fallopian tube cancers (ClinicalTrials.gov Identifier: NCT00108745). A Phase 1 trial is underway to evaluate PEGylated Irinotecan (JK-1201I) in patients with malignant solid tumors (ClinicalTrials.gov Identifier: NCT04366648). PEG-BCT-100 is a novel PEGylated formulation of recombinant human arginase, which can deplete arginine levels and starve cancer cells. It has thus far shown to be safe to use and is entering Phase 2 trials (ClinicalTrials.gov Identifier: NCT03455140).

Polymeric nanoparticles (NPs) are unmatched in versatility, having a plethora of design elements with endless possibilities. Base materials can be synthesized from monomers or biomacromolecules, or a combination of both, with drugs directly conjugated or loaded. Surface charge, size, and density can be modulated to suit applications ranging from drug-loaded hydrogels to core–shell NPs for gene therapy, and fabrication technique can be adjusted according to desired material [26, 43]. Nanoparticle composition can be controlled to best complement the cargo properties and target, incorporating elements to increase biocompatibility, biodistribution, stability, and efficacy [142]. Most importantly, synthetic flexibility of polymer-based NPs allows for built-in functionalities that can enable specific targeting and release [143]. However, with greater intricacy comes greater challenges for manufacturing and uniformity, which is a significant consideration for translation to clinical use [144]. A novel nanoparticle-drug conjugate (EP0057, formerly CLX101/IT-101) composed of a cyclodextrin-based polymer backbone linked to camptothecin (CPT), a topoisomerase 1 (Topo 1) inhibitor, was investigated in a Phase 1b/2 trial in patients with epithelial ovarian cancer (ClinicalTrials.gov Identifier: NCT02389985) and currently a Phase 1/2 trial for lung cancer treatment combined with olaparib (ClinicalTrials.gov Identifier: NCT02769962). CPT stabilizes the Topo 1-DNA cleavage complex during DNA replication and prevents Topo 1 mediated DNA re-ligation, ultimately leading to apoptosis [145]. In preclinical studies, EP0057 induced down-regulation of HIF-1α, a transcription factor associated with angiogenesis, metastasis, and vascular endothelial growth factor (VEGF) inhibitor resistance, and was also shown to accumulate preferentially in human tumor tissue and not in adjacent tissue [145, 146]. Upon evaluation of PK properties, the nanoparticle formulation exhibits high plasma drug retention, slow clearance, and controlled slow release of CPT from the polymer when administered alone and with PTX [147]. Somatostatin receptors (SSRs) are overexpressed in colorectal cancer cells, and currently a Phase 1 clinical trial is underway to investigate ethylcellulose polymeric NPs loaded with Cetuximab and decorated with octreotide, a SSR agonist, to induce specific targeting to colorectal cancer cells [148]. The novel formulation will release Cetuximab at pH 6.8 but is stable at pH 1.5, protecting the stomach and decreasing overall toxicity (ClinicalTrials.gov Identifier: NCT03774680).

Proteins have been widely used for drug delivery systems and diagnostic purposes. Intrinsic properties of proteins such as biocompatibility and biodegradability are highly desirable for nanoformulation, and specific protein interactions can be utilized for selective targeting or uptake. For example, the selective binding of albumin to membrane-associated gp60 (albondin) on the surface of endothelial cells, initiates internalization and active transportation [149]. Caveolae carry albumin and other plasma constituents to the extravascular space of tumors, where further interaction with osteonectin results in accumulation of albumin-bound drugs in the tumor interstitial space, therefore making albumin an excellent vehicle for targeted delivery of anticancer drugs [150]. Abraxane® is an FDA-approved albumin-nanoparticle formulation of PTX which utilizes this mechanism as first line treatment for metastatic breast cancer, advanced non-small cell lung cancer (NSCLC) and late-stage (metastatic) pancreatic cancer [151]. Celgene corporation, the manufacturer of Abraxane®, has developed several albumin-bound therapeutics, with albumin-bound rapamycin (ABI-009) under current investigation in combination with Bevacizumab and mFOLFOX6 in patients with advanced or metastatic colorectal cancer (ClinicalTrials.gov Identifier: NCT03439462). INNO-206, an albumin-DOX conjugate has completed multiple clinical trials, and is now being evaluated as part of combination therapy against locally advanced or metastatic pancreatic cancer (ClinicalTrials.gov Identifier: NCT04390399).

Antibody–drug conjugates (ADCs) have been gaining momentum for cancer therapies, with several FDA approved within the last few years [152]. They target specific antigens that are overexpressed on tumor cells but minimally expressed in healthy cells and deliver a cytotoxic drug upon cellular uptake and subsequent cleavage of a linker molecule [153]. ADCs have several advantages including minimal immunogenicity, prolonged half-life of cytotoxic drugs, and efficient receptor-mediated endocytosis. The linker is a critical point of design for ADCs since it must be stable enough to keep the ADC intact while in circulation and labile enough to release the payload at the target site [66, 154]. Cleavable linkers can be beneficial as they can be tuned to specific environmental stimuli to release the drug from antibody, while non-cleavable linkers are more stable while circulating and depend on antibody degradation. Site-specific conjugation further contributes to PK/PD parameters and stability [155]. There are over 100 ADCs undergoing active clinical trials, and several that were FDA approved within the last two years, revealing next-generation ADCs with optimized linkers.

Diffuse large B-cell lymphoma (DLBCL) is the most frequent form of the aggressive non-Hodgkin lymphoma, accounting for approximately 30–58% of cases, and long-term survival is rare, thus new therapies are in high demand [156]. Earlier this year, Zynlonta (loncastuximab tesirine) was approved for relapsed or refractory DLBCL, including patients who failed to respond to CAR-T therapy, which accounts for approximately 40–50% of patients [157]. In 2020, Trodelvy (sacituzumab govitecan) was approved by the FDA for triple negative breast cancer (TNBC) [158]. Trodelvy targets Trop-2, which is over-expressed on TNBC, using a mAb and a proprietary hydrolysable linker to deliver the cytotoxic payload SN-38 to tumors. This linker, importantly, also creates an effective bystander effect in the tumor micro-environment and can deliver large quantities of SN-38 directly to tumors [159]. CAFs exist in the TME and are known to promote angiogenesis, tumorigenicity, and metastatic dissemination of cancer cells [99]. CAFs express fibroblast activation protein (FAP), a type II transmembrane protein overexpressed in over 90% of colon, breast, and lung cancer CAFs [160]. Antibody-conjugated drug Enfortumab Vedotin targeting FAP-positive CAFs was highly effective in clinical trials for advanced bladder cancers and was awarded FDA approval in 2019. Three other ADCs were approved in 2019, Polivy(polatuzumab vedotin-piiq) for DLBCL, Padcev (enfortumab vedotin) to treat locally advanced or metastatic urothelial cancer, and Enhertu (trastuzumab deruxtecan) to treat HER-2 + breast cancer and gastric or gastroesophageal junction (GEJ) adenocarcinoma [161].

The oncofetal tumor-associated antigen 5T4 has been linked with cancer stem cell properties in multiple cancer types and is associated with the spread of tumors [162]. Furthermore, the 5T4 protein is expressed by many different cancers but rarely in normal adult tissues, making it an attractive candidate to improve specificity for cancer therapeutics [163]. There are currently clinical trials underway with therapeutics targeting the 5T4 antigen (ClinicalTrials.gov Identifier: NCT04202705). SYD1875 is a next generation ADC, comprised of a humanized IgG1 monoclonal antibody targeting the 5T4 oncofetal antigen, and a cleavable linker-drug called valine-citrulline-seco-DUocarmycin-hydroxyBenzamide-Azaindole (vc-seco-DUBA), employing site specific conjugation that improves efficacy, exposure, and manufacturing process [164]. This proprietary ADC utilizes an inactivated synthetic duocarmycin-based cytotoxin that rapidly decomposes if released prematurely, further demonstrating its specificity and stability [155]. A similar next-generation ADC (SYD985) targeting HER2 received fast track designation from the FDA and is currently in a pivotal Phase 3 clinical trial for locally advanced or metastatic breast cancer (ClinicalTrials.gov Identifier: NCT03262935). It is also in two Phase 2 clinical trials for early-stage breast cancer (NCT01042379), advanced or metastatic endometrial cancer (NCT04205630), and Phase 1 trial in combination with the PARP inhibitor niraparib in patients with solid tumors (NCT04235101).

#### Nanocarriers for gene therapy

Gene therapy is a major player in the fight against cancer, delivering nucleic acids to express pro-apoptotic proteins, substitute mutated genes, down-regulate or silence oncogenic pathways, produce anti-cancer cytokines, and/or activate the immune system against cancer [165]. One of the major challenges of gene delivery is successful delivery of nucleic acids to the target site while avoiding degradation. In 2019, Patisiran (ONPATTRO®) was the first siRNA-delivering liposome to be FDA approved, delivering siRNA against the gene responsible for transthyretin protein expression, which can cause hereditary transthyretin amyloidosis. Efficient and safe delivery methods for gene therapy continue to present challenges for clinical translation. Recombinant viral vectors are superior to nonviral vectors with regards to gene delivery, but also come with limitations such as immune response, large-scale manufacturing, gene size limitation, narrow cell tropisms, and lack of surface modifiability without compromising vector integrity [166]. Non-viral vectors are synthetically dynamic, exhibit low immunogenicity, and have simpler large-scale production, but can have reduced transfection capability compared to viral vectors. Recently, two vaccines against the SARS-CoV-2 virus utilizing adenovirus vectors have been linked to several cases of thrombotic thrombocytopenia but are still under scientific investigation, while interestingly, the Moderna and Pfizer/BioNTech vaccines employing lipid-based carriers demonstrate higher efficacy and no link to thrombotic complications [167, 168]. Continued development of inert and efficient nanocarriers for nucleic acid-based cancer therapies remains a priority, and there are several currently being tested in clinical trials.

Polo-like kinase 1 (PLK1) is overexpressed in a multitude of human cancers, and inhibition of PLK1 can induce mitotic arrest and apoptosis, indicating utility for siRNA to silence PLK1. Stable nucleic acid lipid particles (SNALPs) are composed of a high transition temperature phospholipid, a PEGylated lipid, and an ionizable cationic phospholipid [169]. The result is high encapsulation efficiency, with neutralization of the net surface charge upon nucleic acid encapsulation, creating more stable vesicles than conventional cationic liposomes. TKM-080301 is a SNALP formulation containing siRNA against the PLK1 gene currently being studied for use in patients with primary or secondary liver cancer (ClinicalTrials.gov Identifier: NCT01437007). In previous clinical studies, TKM-080301 was generally well-tolerated by solid tumor patients and demonstrated a preliminary antitumor efficacy (ClinicalTrials.gov Identifier: NCT02191878).

Eph receptor A2 (EphA2) is part of the receptor tyrosine kinase family that modulates cell differentiation, survival, and proliferation, and it is overexpressed in multiple cancer types [170]. A Phase 1 trial is currently evaluating 1,2-Dioleoyl-sn-glycero-3-phosphocholine (DOPC)-liposomes delivering EphA2 siRNA in treating patients with advanced and/or recurrent solid tumors (ClinicalTrials.gov Identifier: NCT01591356). The transforming growth factor-β (TGF-β) is a family of structurally related proteins that control numerous cellular functions including proliferation, apoptosis, differentiation, epithelial-mesenchymal transition (EMT), and migration [171]. It has been implicated in tumor promoting effects, particularly in late stages of several cancer types. STP705 is a proprietary polypeptide nanoparticle delivering siRNA against both TGF-β1 and cyclooxygenase-2 (COX-2) [172]. COX-2 is also overexpressed in many types of cancers, promoting carcinogenesis, and inducing resistance to both chemo- and radiotherapies. STP705 is currently being investigated as gene therapy for cutaneous squamous cell carcinoma, hepatocellular carcinoma, and basal cell carcinoma (ClinicalTrials.gov Identifier: NCT04844983, NCT04676633, NCT04669808).

For treatment of NSCLC, GPX-001 (quaratusugene ozeplasmid) is a lipid nanoparticle delivering the gene for TUSC2, a protein which elicits anti-tumor effects through regulating G1 cell cycle progression, apoptosis, calcium homeostasis, gene expression, and tyrosine and Ser/Thy kinase activity [173]. Gene carriers delivering TUSC2 have been shown to interrupt cell signaling pathways that cause replication and proliferation of cancer cells, re-establish pathways for apoptosis, block drug resistance mechanisms, and modulate the immune response against cancer cells [174, 175]. In January 2020, the FDA granted Fast Track Designation for GPX-001 for NSCLC in combination therapy with osimertinib for patients with EFGR mutations whose tumors progressed after treatment with osimertinib alone (ClinicalTrials.gov Identifier: NCT04486833).

Rexin-G was the first targeted gene therapy vector to gain fast track designation and orphan drug priorities for multiple cancer indications in the US. Rexin-G is a replication-incompetent retroviral vector utilizing a cryptic collagen-binding motif on its envelope for targeting abnormal Signature (SIG) proteins in tumors (ClinicalTrials.gov Identifier: NCT00504998) [176]. Abnormal collagenous SIG proteins are a consequence of tumor invasion, angiogenesis, and stroma formation, thus targeting will induce vector accumulation within the TME [177]. CCNG1 gene expression is highly involved in cell cycle regulation, and is tightly associated with oncoproteins such as Mdm2 and cMyc, and the p53 tumor suppressor protein [178]. CCNG1 is overexpressed in over 50% of various malignancies, including pancreas, breast, prostate, ovarian, and colon cancer [179]. Rexin-G encodes a dominant-negative mutant construct (dnG1) of human cyclin G1 (CCNG1) to produce a cytocidal dnG1 protein that effectively blocks a pivotal checkpoint of the cell division cycle, resulting in apoptosis. Rexin-G was shown be exceptionally safe and exhibit dose-dependent antitumor activity in patients with gemcitabine-refractory metastatic pancreatic adenocarcinoma [176]. NG-641 is an oncolytic adenoviral vector encoding four genes: a bi-specific FAP-targeted T-cell activator to activate T-cells to kill fibroblasts, plus three additional genes to further recruit and activate those T-cells (CXCL9, CXCL10, and interferon alpha) [180]. A phase 1, first in-human study is underway to evaluate safety and tolerability combination with nivolumab in patients with metastatic or advanced epithelial tumors and to determine the recommended dose (ClinicalTrials.gov Identifier: NCT05043714). Another first in-human study is beginning for rQNestin34.5v.2, an oncolytic viral vector made from the herpes simplex virus type 1 (HSV1). In some cases, HSV1 can cause severe infection of the brain and liver and/or death, however the rQNestin virus has been modified to replicate only in glioma cells but not in normal, healthy cells [181]. The UL39 gene encoding the viral ribonucleotide reductase large subunit infected cell protein 6 (ICP6) and both endogenous copies of the gamma34.5 gene that encodes for the RL1 neurovirulence protein infected cell protein 34.5 (ICP34.5) (needed for robust viral growth in an infected cell) are deleted, and one copy of the gamma34.5 gene is reinserted under control of a nestin promoter, which is selectively activated in gliomas [182]. By inactivating UL39, viral ribonucleotide reductase activity is disrupted, resulting in the inhibition of nucleotide metabolism and viral DNA synthesis in non-dividing, healthy cells but not in dividing cells [183]. This clinical study will determine the safety and dosing of rQNestin (ClinicalTrials.gov Identifier: NCT03152318). AAV2hAQP1, utilizes an adeno-associated viral (AAV) vector to encode human aquaporin-1 to one parotid salivary gland. Though not directly used to treat cancer, it is currently being tested to alleviate severe dry-mouth associated with radiation therapy [184]. After testing with an adenovirus vector, which demonstrated efficacy but some immunogenicity, it is expected that the AAV vector can safely transfer the human aquaporin-1 (hAQP1) cDNA gene to parotid glands of adult patients with IR-induced salivary hypofunction to elevate salivary output (ClinicalTrials.gov Identifier: NCT02446249).

Exosomes are 30–100 nm in diameter and contain DNA, miRNA, mRNA, lncRNA, proteins, and other cellular components within their lipid bilayer membrane [185]. Exosomes can enter recipient cells via membrane fusion, and induce transcriptional and translational changes [186, 187]. They are highly biocompatible and stable, exhibit tumor homing, and can be modified, thus hold great potential for cancer therapy [188]. Exosomes derived from normal fibroblast-like mesenchymal cells were engineered to carry siRNA or shRNA specific to oncogenic KRASG12D (iExosomes), a common mutation in pancreatic cancer (ClinicalTrials.gov Identifier: NCT03608631). Compared to liposomes, iExosomes target oncogenic Kras with an enhanced efficacy that is dependent on CD47, and is facilitated by micropinocytosis [189]. iExosomes treatment suppressed cancer in multiple mouse models of pancreatic cancer and significantly increased their overall survival. This phase I trial studies the best dose and side effects of mesenchymal stromal cells-derived exosomes with KrasG12D siRNA (iExosomes) in treating participants with pancreatic cancer with KrasG12D mutation that has spread to other places in the body.

#### Immunotherapeutic applications of nanotechnology

Breakthrough achievements have been made in the realm of immunotherapies for cancer including CAR-T cell therapy, immune checkpoint inhibitors, and cancer vaccines. The guiding principle behind immunotherapy is recognition of tumor-associated antigens (TAAs) or tumor-specific antigens (TSAs) by the adaptive immune system [190]. TAAs can be found across all cell types, but are typically overexpressed in tumor cells while TSAs are present only in tumor cells [191]. In order to generate tumor-directed immune responses, the tumor-associated protein is taken up by antigen-presenting cells (APCs) and processed in small protein fragments [192]. After binding to patient-specific human leukocyte antigen (HLA) molecules, the HLA-peptide complex is recognized by the T cell receptors (TCR) and upon binding, the T cell induces tumor cell death [193].

Some TAAs can come from reactivation of embryonic genes which are normally found in differentiated cells, and New York esophageal squamous cell carcinoma 1 **(**NY-ESO-1) is a cancer-testis antigen normally expressed in testicular germ cells and trophoblasts of the placenta [194]. NY-ESO-1 is also expressed in a wide range of cancers with a high incidence (around 20–40% of several advanced cancers, such as melanoma [46%], round cell liposarcoma [89–100%], neuroblastoma [82%], and ovarian [43%] cancer). The NY-ESO-1 antigen has been used in dozens of clinical studies, inducing an improved immune response and positive outcomes in certain trials thereby confirming its utility for cancer therapy. Invariant natural killer T (iNKT) cells are a subset of immune cells that recognize glycolipid antigens presented by the non-polymorphic MHC class I-like molecule, CD1d. [195, 196]. Upon activation they efficiently produce cytokines that stimulate other immune cells and boost cytotoxic T cell responses, and iNKT agonists have high adjuvant effects when administered simultaneously, even at low doses [197, 198]. Poly(lacto-co-glycolic acid) (PLGA) is a biodegradable polymer with minimal (systemic) toxicity, approved by the FDA and the European Medicines Agency (EMA) for use in various drug-carrying platforms. PLGA-based NPs containing the tumor antigen NY-ESO-1 and the iNKT cell activator IMM60 are currently in a Phase 1 clinical trial to test anti-tumor responses in cancer patients (ClinicalTrials.gov Identifier: NCT04751786). Encapsulating antigens and adjuvants within the same polymeric nanoparticle can enhance T cell responses [199]. In earlier studies, the NY-ESO-1 whole protein was encapsulated in adjuvant ISCOMATRIX and shown to induce specific T cell responses in a majority of patients [200]. Previous clinical trials have already shown the safety and tolerability of the NY-ESO-1 protein and peptides in patients with advanced cancer.

To facilitate NY-ESO-1 antigen encapsulation, long (85–111(peptide #2) and 117–143(peptide #3)) and short (157–165(peptide #4)) peptides are incorporated into NPs. Similar peptides (79–116 and 118–143) were previously loaded onto DCs together with α-GalCer and delivered to cancer patients in a recent clinical trial. The results of that trial demonstrated iNKT cell expansion, CD4 + T cell responses against the 118–143 peptide in 7/8 patients, and CD8 + T cell responses against the 79–116 peptide in 3/8 patients [201]. Here, an additional short peptide (157–165) is included, which is presented by the highly prevalent HLA-A2.1 molecule. Hence, higher CD8 + T cell responses against this epitope and superior activation of human iNKT cells by IMM60 are expected due to co-encapsulation [199, 202].

mRNA cancer vaccines are an emerging asset in the fight against cancer, designed to work against TSAs [203]. These antigens can be identified quickly through next-generation sequencing and bioinformatics tools, and engineered into mRNA vaccines, which have recently taken limelight with the success of mRNA SARS-CoV-2 vaccines. mRNA vaccines have considerable advantages over DNA vaccines including higher levels of protein expression, fast and temporary protein expression, simpler manufacturing process, and no genomic integration [203, 204]. However, nucleic-acid based therapies are subject to swift degradation and insufficient cellular uptake, therefore nanoformulation is essential for proper delivery [165]. Both Moderna and BioNTech have developed promising nanoformulated, mRNA-based cancer vaccines, and are currently being tested in clinical trials [203]. Moderna’s personalized cancer vaccines are derived from individual tumor sequencing to elicit a more effective anti-tumor response against TSAs [205, 206]. A single vaccine may deliver mRNA encoding up to 34 unique TSAs, pushing therapeutics into the next era of personalized medicine [207]. In the current trial, mRNA-4157 coated with lipid NPs is given alone to participants with resected solid tumors and in combination with Pembrolizumab in participants with unresectable solid tumors (ClinicalTrials.gov Identifier: NCT03313778). Interim data showed that mRNA-4157 given in combination with Pembrolizumab is well tolerated at all dose levels and produced responses as measured by tumor shrinkage by in human papillomavirus (HPV)(-) head and neck squamous cell carcinoma (HNSCC) patients [208].

The Lipo-MERIT trial is the first in-human testing an mRNA vaccine (BNT111/Melanoma FixVac), a liposomal formulation of mRNA encoded against four distinct malignant melanoma-associated antigens: NY-ESO-1, melanoma-associated antigen A3, tyrosinase, and transmembrane phosphatase with tensin homology (ClinicalTrials.gov Identifier: NCT02410733). In preclinical murine studies, the RNA-lipoplexes were engineered to target dendritic cells (DCs) by altering the lipid:RNA ratio, and they effectively transfected splenic antigen-presenting cells, activated NK, B, CD4 + , CD8 + T cells, and produced interferon alpha (IFN-α).) [209]. An exploratory interim analysis showed that vaccine, alone or in combination with blockade of the checkpoint inhibitor PD1, mediates durable objective responses in checkpoint-inhibitor (CPI)-experienced patients with unresectable melanoma. [210]. Clinical responses were accompanied by the induction of strong CD4 + and CD8 + T cell immunity against the vaccine antigens. Further FixVac cancer vaccine candidates are currently investigated in Phase 1 clinical trials for prostate cancer (BNT112) (Clinicaltrials.gov Identifier NCT04382898), HPV16-positive cancers (BNT113) (Clinicaltrials.gov Identifier NCT03418480), triple negative breast cancer (BNT114) (Clinicaltrials.gov Identifier NCT02316457) and ovarian cancer (BNT115) (Clinicaltrials.gov Identifier NCT04163094). The first-in-human, open label Phase 1 study is underway to investigate a liposomal mRNA vaccine (W_ova1 vaccine) delivering three ovarian cancer TSA RNAs in ovarian cancer patients, where patients will be vaccinated intravenously prior, and during (neo)-adjuvant chemotherapy (ClinicalTrials.gov Identifier: NCT04163094). Overall, mRNA vaccines formulated in nanocarriers have shown initial clinical promise by targeted delivery to APCs. These nanovaccines are standalone immunotherapeutics that activate the immune system against specific antigens and have also been combined with checkpoint antibodies in several recent trials, which are expected to achieve better therapeutic outcomes [211].

### Prospective nanotechnologies to advance cancer therapy

**Fig. 6:**
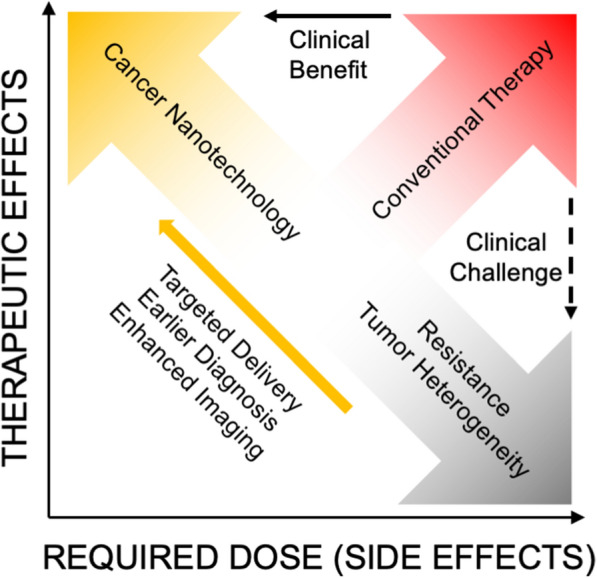
Tumor heterogeneity and drug resistance are two major obstacles that conventional chemotherapy face. Nanotechnology can overcome these obstacles through multi-modal treatments and increase therapeutic efficacy while decreasing dosage by utilizing targeted delivery, stimuli-triggered release, and formulations to improve PK/PD profiles. Adapted with permission, [30] 10.1016/j.addr.2015.10.019

As emerging nanotechnologies seek to improve PK/PD, efficacy, and specificity, many preclinical studies are underway to achieve triggered drug release and multi-modal therapies that will be highly selective toward cancerous cells. Targeted drug release can further decrease minimum required dose and ultimately decrease overall toxicity, improving efficacy and patient quality of life (Fig. 6) [30]. As technology advances to utilize specific delivery, therapeutics can be formulated to achieve optimal efficacy and minimal toxicity.

Certain targeted therapies can exhibit tumor specificity but have clinical limitations due to PK/PD properties or biodistribution. Tumor necrosis factor-related apoptosis-inducing ligand (TRAIL) is an ideal anti-cancer agent because of its potency and specificity toward cancerous cells while leaving healthy cells unaffected. [212]. However, it struggles to move past preclinical because of a short half-life and rapid renal clearance of the off-targeted TRAIL [213]. A new development of a TRAIL-active trimer ferritin nanocage (TRAIL-ATNC) has 16 times longer serum half-life while maintaining anti-tumor efficacy in vivo in xenograft breast cancer and orthotopic pancreatic models [64]. Nanoformulation has the potential to improve PK/PD parameters for any therapeutic, opening the door to drug repurposing [214]. Recently, lipid tail modifications of cationic liposomes were shown to increase the loading capacity of highly hydrophobic PTX helpful for the development of liposomal delivery of PTX to reduce the side effects and cost. It was found that lipid tails containing one oleoyl (DOPC/DOTAP) had lower loading capacity compared to the newly synthesized DLinTAP containing two linoleoyl tails, demonstrating that even minor modifications to nanoformulation can significantly improve drug delivery systems. [122].

Stimuli-responsive carriers are designed to release payload under specific conditions such as changes in pH, changes in temperature, overexpression of specific enzymes found within TME, increased levels of intracellular components such as glutathione, and external stimuli such as radiation, ultrasound, magnetic field, etc. [56]. In this respect, specific delivery can be achieved with drug release within the TME or other desired targeted areas. TP53 is the one of the most frequently mutated or deleted genes in breast cancer, with the mutation observed in up to 44% of TNBC compared with 15% in ER-positive breast cancers [215]. Both the loss of TP53 and the lack of targeted therapy are significantly correlated with poor clinical outcomes, making TNBC the only type of breast cancer that has no approved targeted therapies [216]. pH-activated NPs were used to enhance the bioavailability and improve endo/lysomal escape of POLR2A siRNA for treatment of TNBC, where POLR2A in the TP53-neighbouring region was identified as a collateral vulnerability target in TNBC tumors. [217].

**Fig. 7:**
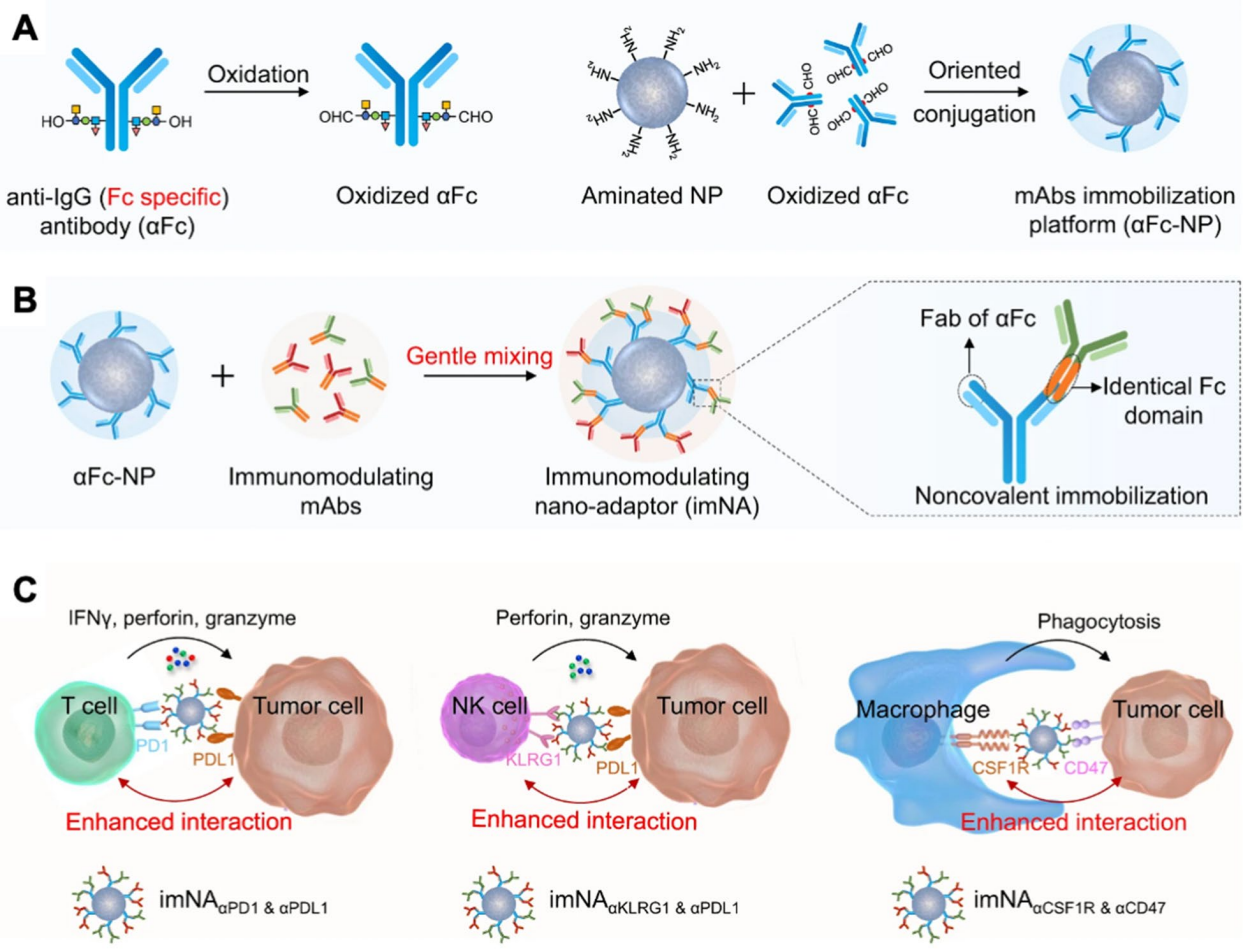
**A** Conjugation of an anti-IgG (Fc specific) antibody (αFc) to nanoparticle (αFc-NP). **B** Two types of immunomodulating monoclonal antibodies (mAbs) targeting effector cells and tumor cells immobilized onto αFc-NP to create immunomodulating nanoadaptors (imNA). **C** imNAs were validated in T cell-, natural killer cell- and macrophage-mediated antitumor immune responses in multiple murine tumor models. Reprinted with permission, [218] 10.1038/s41467-021-21497-6

Cancer immunotherapy currently relies on two major strategies: modulating effector immune cells via monoclonal antibodies (mAbs) and facilitating the co-engagement of T cells and tumor cells via chimeric antigen receptor- T cells or bispecific T cell-engaging antibodies. Integrating the two strategies into one system may be the future of cancer immunotherapy, and it was recently demonstrated in a versatile antibody immobilization nanoplatform constructed by attaching anti-IgG (Fc specific) antibody (αFc) on the surface of a nanoparticle (αFc-NP), allowing two types of monoclonal antibodies to be immobilized (Fig. 7) [218]. Immunomodulating nano-adaptors (imNAs) outperformed a combination of mABs in T cell and natural killer cell, and macrophage driven immune response in multiple murine tumor models.

Novel nanomaterials can further enhance cancer immune therapies, for example outer membrane vesicles (OMVs) are secreted by Gram-negative bacteria, sized 30–250 nm, which serve as a mediator of bacteria communication and homeostasis [219]. They possess intrinsic immunostimulatory properties and have desirable properties for vaccine delivery such as small size and ease of scale-up production [220]. It was recently shown that tumor antigens can be displayed on OMV surfaces as ClyA fusion proteins that can induce T-cell mediated, specific anti-tumor immunity. [221]. Furthermore, using protein “Plug-and Display” technology, a protein tag can spontaneously bind to the protein catcher through isopeptide bond formation. Various tumor antigens linked to protein tags can be rapidly and simultaneously displayed on the OMV surface, and after accumulation in draining lymph nodes, can be processed and presented by DCs [220].

Nanomaterials have shown to be extremely useful for co-delivery of multiple chemotherapeutic agents. Drugs have various biochemical properties that can be drastically different from its synergistic complement, therefore co-delivery within a single carrier can normalize distribution and delivery [222]. Anti-PD-1/PD-L1 antibodies are currently used in the clinic to interrupt the immune checkpoint, which reverses T cell dysfunction/exhaustion and shows success in treating cancer [223]. A liposomal formulation of histone demethylase inhibitor, 5-carboxy-8-hydroxyquinoline (IOX1) and DOX was recently reported to promotes T cell infiltration/activity and significantly reduce tumor immunosuppressive factors. [224]. In vivo studies showed reduced growth of various murine tumors (subcutaneous, orthotopic, and lung metastasis), and offers a long-term immunological memory function against tumor rechallenging. The study showed that IOX1 inhibits cancer cells’ P-glycoproteins (P-gp) through the JMJD1A/β-catenin/P-gp pathway and synergistically enhances DOX-induced immune-stimulatory immunogenic cell death. Nanoformulation can tune release kinetics for dual-drug loading, optimizing drug release depending upon desired outcome [57]. Drug release can occur through various modes of activation; therefore, the release rate can be highly specific to the stimuli-responsive enhancements [71]. Mesoporous silica NPs (MSNs) coated with polyacrylic acid (PAA), and pH-sensitive lipid (PSL) were recently engineered for co-delivery and dual-pH-responsive sequential release of arsenic trioxide (ATO) and PTX (PL-PMSN-PTX/ATO).) [225]. Tumor-targeting peptide F56 was used to modify MSNs, which conferred a target-specific delivery to cancer and endothelial cells under neoangiogenesis. The drug-loaded NPs displayed a dual-pH-responsive (pHe 6.5, pHendo 5.0) and sequential drug release profile. PTX within PSL was preferentially released at pH 6.5, whereas ATO was mainly released at pH 5.0. Drug-free carriers showed low cytotoxicity toward MCF-7 cells, but ATO and PTX co-delivered NPs displayed a significant synergistic effect against MCF-7 cells, showing greater cell-cycle arrest in treated cells and more activation of apoptosis-related proteins than free drugs. Furthermore, the extracellular release of PTX caused an expansion of the interstitial space, allowing deeper penetration of the NPs into the tumor mass through a tumor priming effect. As a result, FPL-PMSN-PTX/ATO exhibited improved in vivo circulation time, tumor-targeted delivery, and overall therapeutic efficacy.

CRISPR-Cas9 gene editing has the potential to permanently disrupt tumor survival genes, which could supersede current limitations and pitfalls of traditional therapies [226]. Several companies are currently developing CRISPR–Cas9 therapeutics, but development of safe and efficient delivery modes remains a need for CRISPR-based therapies to be utilized in clinical applications. A novel amino-ionizable lipid nanoparticle (LNP) was recently formulated for the delivery of Cas9 mRNA and sgRNAs [227]. A single intracerebral injection of CRISPR-LNPs against PLK1 (sgPLK1-cLNPs) into aggressive orthotopic glioblastoma enabled up to ~ 70% gene editing in vivo, which caused tumor cell apoptosis, inhibited tumor growth by 50%, and improved survival by 30%. To reach disseminated tumors, cLNPs were also engineered for EGFR-targeted delivery. Intraperitoneal injections of EGFR-targeted sgPLK1-cLNPs caused their selective uptake into disseminated ovarian tumors, enabled up to ~ 80% gene editing in vivo, inhibited tumor growth, and increased survival by 80%. In another recent study, controlled release of CRISPR-Cas9 ribonucleoprotein (RNP) and codelivery with antitumor photosensitizer chlorin e6 (Ce6) was achieved using near-infrared (NIR)– and reducing agent–responsive NPs in a mouse tumor model [228]. Nitrilotriacetic acid–decorated micelles bound His-tagged Cas9 RNP, and lysosomal escape of NPs was triggered by NIR-induced reactive oxygen species (ROS) generation by Ce6 in tumor cells. Reduction of disulfide bond allowed cytoplasmic release of Cas9/single-guide RNA (sgRNA) targeting the antioxidant regulator *Nrf2*, enhancing tumor cell sensitivity to ROS, and demonstrating synergistic therapy in vivo. A plethora of exciting nanotechnologies exist that substantially improve cancer therapies, but there remain some obstacles for clinical translation such as scalability, homogeneity, and regulatory guidelines.

## Cancer diagnostics on the nanoscale

As the saying goes, “an ounce of prevention is worth a pound of cure”; with regards to cancer treatments, it may be worth one metric ton of cure. Development of cancer pharmaceuticals is a costly endeavor, to say the least. The cost for developing a successful drug can enter the billion-dollar realm, and the majority of drug candidates do not pass clinical trials [229]. Each year, the total oncology pipeline consists of hundreds of molecules in late-stage development, but only 50 new small molecule anti-cancer drugs were approved by the FDA from 2015–2020 [230]. As aforementioned, the prevalence of drug resistance necessitates development of new therapeutics, driving up costs. Conversely, an highly accurate diagnostic test can be indefinitely rewarding and impactful by effectively detecting cancer at early stages, lowering patient costs, and extending survival [231]. Early detection of cancer has major implications for likelihood of treatment success and overall survival statistics since 90% of cancer-related deaths are caused by metastasis. [232]. Even average patient cost is significantly increased for treatment of late-stage cancer diagnosis vs. early stage. [233]. The benefits of early detection and routine screening are innumerable, particularly since certain cancers exhibit symptoms only in late stages, and screening methods can be further utilized to evaluate and optimize treatment specifically to each patient [234, 235]. Although technology has advanced in several areas, the need remains for efficient routine screening methods that can accurately detect any type of cancer at early stages without overdiagnosis [236]. Nanomaterials may meet this need as their unique optical, magnetic, mechanical, chemical, and physical properties can enhance sensitivity and precision for cancer biomarker detection.

### Classic diagnostic techniques

Aside from a few selected cancers which are routinely screened, certain cancer diagnoses occur only after the onset of symptoms, when cancer is typically in later stages [237, 238]. Traditional diagnostic methods rely mostly on classic imaging methods with ultrasound, MRI, CT scans, and X-ray, the Papanicolau test to detect cervical cancer, prostate-specific antigen level detection in blood samples, and occult blood detection for colon cancer [239–242]. However, currently available cancer screenings are typically only available to a subset of patients depending on risk level and/or age, specific for the aforementioned cancers as opposed to comprehensive screening of multiple cancer types [243–245]. A solid-tumor cancer will be detected after there has been a significant physical change to the tissue, leaving a large window of time for the undiagnosed cancer to spread and for survival odds to decrease. A tissue biopsy, which is painful and invasive, is needed to confirm and assess for proper treatment selection. Although some metastatic cancers can be obvious to detect, it is nearly impossible to determine via conventional imaging depending on where metastasis is beginning to occur [246]. Surgical resection is typically the next step, but cannot guarantee complete removal of all cancerous cells, especially when attempting to spare as much healthy tissue as possible, and success is highly dependent on tumor margin [247].

### Nanotechnology-improved diagnostics, imaging, and treatment monitoring

With classic imaging techniques incapable of early diagnosis, nanomaterials can considerably improve tumor detection through tumor targeting and specific intrinsic physico-chemical properties that can enhance signal [81]. Certain nanomaterials can further enable new imaging platforms and techniques that have higher sensitivity and without possible harmful effects [85, 248]. NPs are currently utilized in multiple medical tests and screenings, but with very few clinical applications specific for cancer screening [249]. Nano-sensors are extremely sensitive, specific, and capable of multiple target capture, thus are ideal for blood biomarker screening [250, 251]. Furthermore, the accessibility of genetic sequencing enables efficient, detailed diagnosis/prognosis to optimize the treatment course, and several nanoformulations are currently being studied for clinical use (Table 3).

**Table 3:** Current clinical investigations of nanomaterials used for cancer diagnostics and imaging

NCT Number	Title	Conditions	Interventions	Phases
NCT04825002	Detection of clinically significant prostate cancer using a urinary multimarker sensor	Prostate cancer	Diagnostic Test: Urinary multimarker sensor	Not Applicable
NCT04696744	Prospective Study for the prognostic and predictive role of circulating tumor cells in patients with oropharyngeal advanced squamous cell carcinoma: CTCO (circulating tumor cells in the oropharynx)	Circulating tumor cell/oropharyngeal cancer	Biological: CTC detection	
NCT04661176	Evaluation of the Sentinel™ PCC4 assay for diagnosis, prognosis and monitoring of prostate cancer in puerto rico	Prostate cancer	Diagnostic Test: miR Sentinel™ PCC4 Assay	
NCT04482803	Targeted biopsy of carbon NPs labelled axillary node for cN + breast cancer	Location and Biopsy of Axillary Lymph Nodes	Drug: Carbon NPs suspension injection will be injected into or around the cortex of the clinically assessed positive lymph nodes	Not Applicable
NCT04300673	Radio guided lymph node dissection in oligometastatic prostate cancer patients	Prostate cancer/lymph node metastases	Procedure: Radio guided surgery (RGS) using Indium-labelled PSMA	Phase 1/2
NCT04290923	Determination of blood tumor cells	Leukemia, lymphocytic, chronic, b-cell/abnormalities cell epithelial		
NCT04261777	Ferumoxtran-10-enhanced MRI in prostate cancer patients	Prostate cancer/metastasis/prostatectomy	Drug: Ferrotran® (Ferumoxtran-10)	Phase 3
NCT04239105	Detection of circulating tumor cells in breast cancer patients using a novel microfluidic and Raman spectrum device	Breast neoplasms/circulating tumor cells	Device: Microfluidic and Raman spectrum	
NCT04167969	The use of NPs to guide the surgical treatment of prostate cancer	Prostate cancer	Drug: (64Cu)-labeled PSMA-targeting particle tracer, or 64Cu-NOTA-PSMAi-PEG-Cy5.5-C' dots/Diagnostic Test: PET/MRI/Other: Blood and urine sampling/Procedure: laparoscopic radical prostatectomy and bilateral pelvic LN dissection	Phase 1
NCT04167722	How does prostate cancer metastasize? Studying the role of secreted packages (exosomes) from fat tissue in lean and obese patients	Prostate cancer/obesity	Procedure: Robotic radical prostatectomy	
NCT03967652	Cancer diagnoses from exhaled breath with Na-nose	Volatile organic compounds/cancer/diagnoses disease	Diagnostic Test: Nanomaterial-based sensors	
NCT03817307	Validation of USPIO-enhanced MRI for detection of lymph node metastases in head and neck carcinoma	Head and neck squamous cell carcinoma	Diagnostic Test: USPIO-enhanced MRI	Not Applicable
NCT03694483	Prostasomes as diagnostic tool for prostate cancer detection	Prostate cancer	Diagnostic Test: Genetic analysis for the detection of prostasomes	
NCT03550001	Carbon NPs as lymph node tracer in rectal cancer after neoadjuvant radiochemotherapy	Rectal cancer	Procedure: Injection CNP before NAT	Not Applicable
NCT03427450	Harvest of CTCs from mbc patients using the Parsortix™ PC1 system	Healthy volunteers/breast cancer, metastatic	Diagnostic Test: Blood draw	
NCT03280277	Ferumoxytol-enhanced MRI in imaging lymph nodes in patients with locally advanced rectal cancer	Locally advanced rectal carcinoma/Stage III rectal cancer AJCC v7/Stage IIIA rectal cancer AJCC v7/Stage IIIB rectal cancer AJCC v7/Stage IIIC rectal cancer AJCC v7	Procedure: Contrast-enhanced magnetic resonance imaging/Drug: Ferumoxytol	Early Phase 1
NCT03134846	Image guided surgery for margin assessment of head and neck cancer Using Cetuximab-IRDye800CW conjugate	Head and neck squamous cell carcinoma/margin assessment	Drug: Cetuximab-IRDye800CW	Phase 1/2
NCT02857218	Ferumoxytol-Enhanced MRI in imaging lymph nodes in patients With Stage IIB-IIIC esophageal cancer	Stage IIB esophageal cancer AJCC v7/Stage III esophageal cancer AJCC v7/Stage IIIA esophageal cancer AJCC v7/Stage IIIB esophageal cancer AJCC v7/Stage IIIC esophageal cancer AJCC v7	Drug: Ferumoxytol/Procedure: Magnetic resonance imaging	Early Phase 1
NCT02751606	Nano MRI on 7 Tesla in rectal and breast cancer	Rectal neoplasms/breast neoplasms	Drug: ferumoxtran-10/Device: 7 Tesla MRI/Device: 3 Tesla MRI	Phase 3
NCT02106598	Targeted silica NPs for real-time image-guided intraoperative mapping of nodal metastases	Head and neck melanoma	Drug: fluorescent cRGDY-PEG-Cy5.5-C dots	Phase 1/2
NCT01411904	A novel magnetic needle using iron oxide NPs for the detection of leukemia	Leukemia	Device: MagProbe (TM)	Not Applicable
NCT01359436	e- Ab sensor-based real-time detection of mutant EGFR in clinical specimens from patients of NSCLC	NSCLC (NSCLC)	Device: Electrosensing antibody probing system (e- Ab sensing)	Not Applicable

During cancer dissemination, tumor cell motility and invasiveness increase, enabling tumor cells to enter the bloodstream as CTCs [252]. Eventually, the most aggressive CTCs invade other tissues and form metastatic tumors, resulting in worsened prognosis. Early detection of CTCs can have a tremendous impact on early and accurate diagnosis of cancer, and detailed analyses can identify specific biomarkers to deduce patient prognosis and response to treatment [253, 254]. However, several challenges need addressing for CTC detection to be a reliable clinical diagnostic/prognostic tool for cancer. During early stages of cancer, proportion of CTCs found in circulation is miniscule, and heterogeneity makes them difficult to isolate and analyze [255, 256]. Nanotechnology has several advantages for CTC detection in an accurate, consistent, and robust manner. Various nanostructures have been used for CTC detection including polymeric, magnetic, carbon-based, metal NPs, and quantum dots [87, 257–261].

The epithelial cell adhesion molecule (EpCAM) is a transmembrane glycoprotein that is overexpressed on the majority of primary and metastatic tumors, and is involved in gene regulation, cell proliferation, and cancer cell differentiation and renewal [262]. As aforementioned, CellSearch® system uses EpCAM- targeting magnetic NPs and cell staining to identify CTCs, and there are several currently being tested in clinical trials (ClinicalTrials.gov Identifier: NCT04290923). There are currently two FDA-approved companion diagnostics (Guardant360 CDx and FoundationOne Liquid CDx) that utilize cell-free DNA screening for multiple cancers without nanotechnology enhancement, but there remains a need for accurate early-stage cancer screening. Despite being considered the “gold standard” clinical CTC platform, previous studies have shown that in some diseases such as prostate cancer, CTCs are undetectable in ~ 30% of patients despite the presence of widespread metastatic disease, and particularly CTCs with a purely mesenchymal phenotype [263, 264]. Metastasis has been linked to epithelial to mesenchymal transition (EMT), during which cells undergo morphological changes that induce greater migratory and invasive capabilities and resistance to apoptosis [265, 266]. Microfluidics is another area of technology applicable to CTC detection due to portability, high-throughput capability, and precise control within the microchannel. Certain microfluidics platforms (Parsortix® and Vycap systems) can recover CTCs based upon size and deformability instead of EMT status, and transcriptomic analysis of CTCs can be performed at the scale of a cell after isolation [267, 268]. Transcriptome analysis then provides information on the state of the cell as to its position in the EMT thanks to a molecular signature by phenotype [269]. This highly sensitive and innovative technique will allow the study of the gene expression profile of CTCs, and several devices are currently being tested in clinical trials (ClinicalTrials.gov Identifier: NCT04696744, NCT04239105, NCT03427450).

Screening for prostate cancer relies on the serum prostate-specific antigen test, leading to a high rate of false positives (80%), subsequently unnecessary biopsies and overtreatment [270]. Considering the frequency of the test, there is a critical unmet need of precision screening for prostate cancer. A urinary multi-marker microfluidic biosensor utilizing machine learning is currently under investigation in a Phase 1 clinical trial (ClinicalTrials.gov Identifier: NCT04825002). In preclinical studies, the correlation of clinical state with the signals from urinary multi-markers was analyzed by two common machine learning algorithms [271]. As the number of biomarkers was increased, both algorithms provided a monotonic increase in screening performance. Under the best combination of biomarkers, the machine learning algorithms screened prostate cancer patients with more than 99% accuracy using 76 urine specimens. A novel and emerging approach to diagnostics involves investigating exosomes secreted by various cell types and their association with cancer progression [272, 273]. Since tumor cells secrete exosomes more abundantly and have been implicated in tumorigenesis, metastasis, and TME formation, they are a target for liquid biopsy development [274]. Obesity is prevalent among many populations, and has strong correlation with aggressive prostate cancer and metastasis, though the exact mechanism is still being explored [275]. A current clinical study is underway to analyze exosomes excreted from fat tissue in lean and obese patients who are currently undergoing radical prostatectomy (ClinicalTrials.gov Identifier: NCT04167722, NCT03694483).

The success of diagnostic screening devices relies heavily on non-invasiveness and patient compliance. Testing urine, saliva, or breath are non-invasive, particularly when compared to biopsies or blood draws [276]. Exhaled breath contains minute concentrations of volatile organic compounds (VOCs), even in the healthy state, but in a diseased state the concentrations and composition can distinguish the type and phase of cancer [277, 278]. Na-nose is a nanosenor array utilizing gold nanomaterials to capture and detect VOCs [279]. Chemical interactions between VOCs and gold particles occur at the nanosensor surface, and electron density change causes a maximum shift in the surface plasmon absorption [280]. The gold NPs can also be conjugated with organic molecules for the capture of VOCs, then analyzed using gas chromatography and mass spectrometry. The Na-nose has the advantages of low cost, easy to use, good reproducibility, and real-time detection for large scale clinical application (ClinicalTrials.gov Identifier: NCT03967652).

Chronic infection with oncogenic HPV is the prominent cause of cervical cancer, followed by clonal progression of infected epithelium to cervical precancer, then further invasion [281]. Over 200 different HPV subtypes have been identified, with a subset of these being classified as high risk for oncogenesis [282, 283]. An electrosensing antibody probing system (e- Ab sensor), is currently testing in clinical trials the interaction kinetics between anti-high-risk HPV and its antigen (high-risk HPV) present in patients (ClinicalTrials.gov Identifier: NCT01359436). It uses engineered semiconductive antibodies or virus in vertical and lateral chip (eAbchip) or lateral flow through (eAbsignal) formats. Semiconductive antibodies are bound as a suitable electrosensing probe, which specifically and selectively binds targeted molecules (high-risk HPV) in the test specimens [284]. From assessment of the electric signature of semiconductive anti- high-risk HPV antibodies, the eABprobe could offer sensitive detection and precise quantification of high-risk HPV, thus providing an efficient and accurate screening for cervical cancer.

In addition to early detection of cancers, it is equally important to detect minimal residual disease (MRD) to help predict outcome, identify high risk patients, and monitor treatment efficacy. A concerted effort to increase test sensitivity and accuracy for both early detection and MRD can make a significant impact on treatment course and overall patient survival [285]. Traditionally, leukemia and lymphoma cells are detected through morphological analysis, immunohistochemistry, antibody microarrays, flow cytometry using fluorescent markers, fluorescence in situ hybridization, PCR, and DNA sequencing [286]. Because these cancer types are extremely common and aggressive, effective treatment depends greatly on the accuracy and sensitivity of diagnosis. Signal amplification coupled with NPs may be a viable approach for earlier detection. To improve the detection of leukemia cells in the marrow, antibodies against the acute leukemia antigen CD34 were conjugated to SPIONs and coupled with a “magnetic needle” biopsy (ClinicalTrials.gov Identifier: NCT01411904). In preclinical studies, leukemia cell lines expressing high or minimal CD34 were incubated with anti-CD34-conjugated SPIONs [287]. Microscopy, Superconducting Quantum Interference Device (SQUID) magnetometry, and in vitro magnetic needle extraction were used to assess cell sampling, finding anti-CD34-conjugated NPs preferentially bind high CD34-expressing cell lines. Furthermore, the magnetic needle enabled identification of both cell line and patient leukemia cells diluted into normal blood at concentrations below those normally found in remission marrow samples. Finally, the magnetic needle enhanced the percentage of lymphoblasts detectable by light microscopy by ten-fold in samples of fresh bone marrow aspirate. This signal amplification can have positive impact on MRD detection, thus allowing oncologists to optimize treatment course.

Following initial treatment regimens, cancer patients can relapse with local and/or distant recurrence, with certain cancers at higher risk than others. The metastasis of a lymph node (LN) indicates systemic disease with increased risk of progression, thus detection of LN metastasis can have tremendous impact on prognosis and treatment course [288, 289]. In the past, prostate cancer patients with LN metastasis have had poor prognoses due to inaccurate staging techniques and toxic treatment regimens such as radiotherapy [290, 291]. Radiotherapy of LN metastases also has limitations with a high percentage of patients having metastatic LN outside the routine radiation field [292, 293]. Conventional imaging techniques using CT and MRI are also not sensitive enough to detect a comprehensive total of LN metastases for certain cancers such as prostate cancer [294]. As a result, there is a need to improve lymph node tracers to help improve the amount of lymph node harvest as well as determine the extent of micro-metastases [295]. Sentinel lymph node (SLN) mapping is used in various cancer types, which relies on specific pattern of lymph drainage away from the tumor, therefore if the SLN, or first node, is negative for metastasis, then the nodes after the SLN should also be negative [296]. Indocyanine green (ICG) is fluorescent dye used to identify the lymphatic channels and decipher which nodes to remove [297]. By doing so, patients avoid a complete lymphadenectomy, however disease must be thoroughly staged for accurate prognosis and determination of appropriate treatment approach. Several clinical studies are currently underway to investigate different nanomaterials as lymph node tracers such as fluorescent cRGDY-PEG-Cy5.5-C quantum dots (ClinicalTrials.gov Identifier: NCT02106598), carbon NPs (NCT03550001, NCT04482803), and silica NPs (NCT04167969).

Despite advancements in traditional imaging devices regarding both preoperative diagnostics and staging, there remains room for improvement regarding sensitivity, resolution, and intraoperative procedures. Progress continues for enhancing image-guided surgeries by incorporating specific targeting, optically-active materials, and nano-sized probes for alternative modes of imaging [298, 299]. Nano-enhanced imaging has potential to drastically improve early-stage detection of metastases and residual tumor cells to improve patient prognoses. Ferumoxtran-10, an ultrasmall superparamagnetic iron oxide (USPIO) particle has proven to be a valuable contrast agent for detecting lymph node metastases using a 1.5 Tesla or 3 Tesla MRI scanner in various types of cancer [300]. It is currently being studied in several clinical trials for SLN mapping, including to improve the resolution and sensitivity of nano-MRI by using a 7 Tesla scanner, particularly for small lymph nodes (ClinicalTrials.gov Identifier: NCT03280277, NCT04300673, NCT03817307, NCT02857218, NCT04261777). A precision nano-enhanced approach for monitoring disease progression utilizes triggered aggregation in the TME (Target-Enabled in situ Ligand Assembly [TESLA]) [301]. The particles are built in situ at tumor sites from precursors containing specific moieties which can form larger NPs only after being cleaved by enzymes specific to cancer cell apoptosis. The NPs carry various image contrast agents for monitoring tumor therapy response to optimize effective dosing regimens, and TESLA is currently being investigated for rectal and breast cancer (ClinicalTrials.gov Identifier: NCT02751606).

The margin status of a tumor remains the main prognostic factor after surgical resection in HNSCC [302]. Margin sizes are used to determine adjuvant therapy or need for re-operation, and currently no technology is available in the operating room which reliably supports tumor excision in terms of margin status [303]. In fact, surgeons can only combine pre- operative imaging data with tactile and visual information during surgery for assessing tumor margins with limited accuracy. Near infrared (NIR) fluorescent optical contrast agents can be coupled to targeted compounds to create highly specific and well-resolved image-guided assessment of tumor margins [304]. Tracers utilize antibodies directed against VEGF-A (bevacizumab-IRDye800CW) for breast cancer or against EGFR, (cetuximab-IRDye800CW) for malignant glioma and pancreatic cancer (ClinicalTrials.gov Identifier: NCT01508572, NCT02855086, NCT02736578). First trials have shown that systemic administration of these compounds is safe and tumor specific. Clinical trials for the intraoperative assessment of tumor margins during surgical treatment of HNSCC and esophageal squamous cell carcinoma are currently underway using cetuximab-IRDye800CW (ClinicalTrials.gov Identifier: NCT03134846, NCT04161560).

### The future of cancer diagnostics and imaging

As previously discussed, non-invasive, sensitive methods for cancer screening will be the key to clinically relevant diagnostics. Ultrasmall gold nanoclusters (AuNCs) have been found to make excellent probes for in vivo imaging because of their accumulation at tumor sites and efficient clearance via urine [305]. Multifunction protease nanosensors that react in the cancer cell microenvironment produce a colorimetric signal that could be monitored via urine. It was found in collected urine samples from colorectal cancer mouse models that tumor affected mice had a 13-fold increase in signal compared to healthy mice. Furthermore, novel imaging agents with better sensitivity and specificity can improve early detection during routine screening and help with tumor margin visualization during surgical resection. Recently, optical properties were investigated for multiple dyes and pigments used in tattoo inks, foods, drugs, and cosmetics already FDA approved [306]. Absorption, fluorescence, and Raman scattering properties were evaluated, and several exhibit a multitude of useful optical properties, outperforming some of the clinically approved imaging dyes on the market. The best performing optical inks (Green 8 and Orange 16) were formulated into liposomal NPs to assess their tumor targeting and optical imaging potential in mouse xenograft models of colorectal, cervical and lymphoma tumors. After intravenous injection, fluorescence imaging revealed significant localization of the new “optical ink” liposomal NPs in all three tumor models as opposed to their neighboring healthy tissues (p < 0.05). Nanoformulations of highly sensitive imaging contrast agents have potential to greatly improve cancer imaging, diagnosis, and surgical removal of tumor tissue.

Nanotechnology has greatly impacted the realm of genetic sequencing through various nanopore-based systems, and subsequently, the realm of disease screening. The single molecule real time sequencing (SMRT) system is based on a single DNA polymerase within 60–100 nm cavities prepared by electron beam lithography on a thin aluminum 100 nm sheet deposited on a silica substrate [307]. This technique allows for optical monitoring of DNA sequence with use of fluorescent nucleotides added to the complement strand. Oxford Nanopore relies on passing a single DNA molecule through a nano-sized protein pore set within an electrically-resistant polymer membrane, where each DNA nucleotide base causes specific disruption in the current passing across the membrane [308]. Although both techniques present immense utility for omics data collection, circulating tumor DNA (ctDNA) analysis remains a challenge [309]. Recently, a new method using statistical analysis of the length of time for genetic code to unzip and blocking of the current has shown promise in identifying the precise position of genetic mutations [310]. This proof-of-concept study was demonstrated on oligonucleotides and is being further developed for liquid biopsies. Alternatively, targeted extracellular vesicle (EV) capture holds promise for liquid biopsy development since miRNA, mRNA, and proteins in/on EVs represent potential cancer biomarkers [311]. A high-throughput nano-biochip (HNCIB) for high-efficiency, targeted EV capture was recently developed using total internal reflective fluorescence microscopy for detection. HNCIB detected an up-regulated expression of programmed death-ligand 1 mRNA and protein and miR-21 in EVs derived from patients with lung adenocarcinoma compared to those from healthy donors. In addition to its high-throughput capabilities, it has low sample requirement and fast assay time. EV monitoring has further been useful for drug treatment monitoring effects, which was previously limited to invasive tissue biopsies and complex processes to analyze drug-target interactions. EV monitoring of small-molecular chemical occupancy and protein expression (ExoSCOPE) measures changes in drug occupancy and the composition of proteins present of in small volumes of blood to assess diseases status and success of targeted treatments [312]. It measures changes in drug occupancy and protein composition in molecular subpopulations of extracellular vesicles, and when used to monitor various targeted therapies, the ExoSCOPE revealed EV signatures that closely reflected cellular treatment efficacy. Using a small volume of blood, the ExoSCOPE accurately classified disease status and rapidly distinguished between targeted treatment outcomes, within 24 h after treatment initiation.

**Fig. 8:**
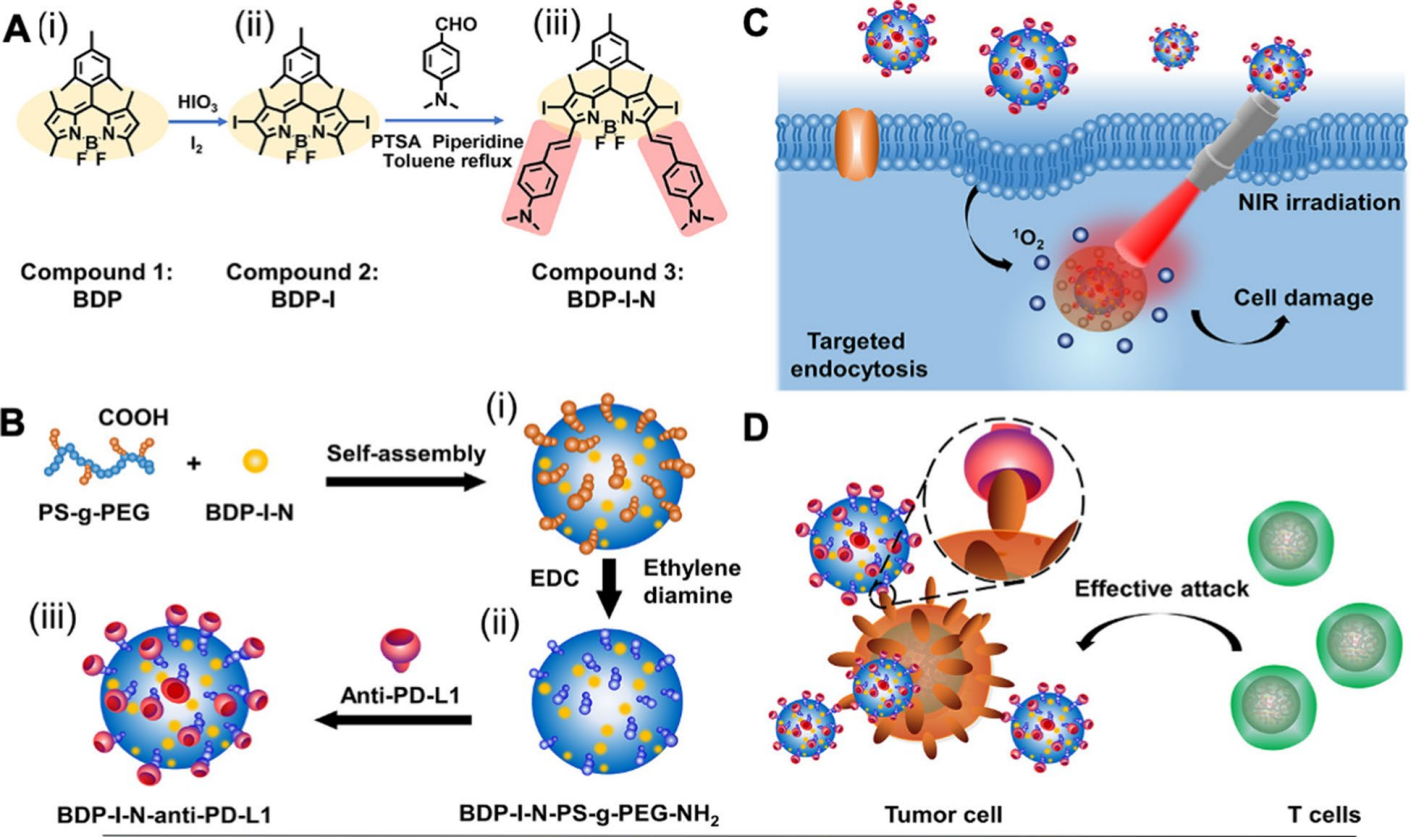
**A** and **B** Synthesis of boron-dipyrromethene compounds and subsequent assembly of nanocarriers functionalized with cell death-ligand 1 (PD-L1) monoclonal antibody. **C** and **D** Schematic of the BDP-I-N-anti-PD-L1-mediated phototoxicity and immune efficacy for tumor cells. Reprinted with permission, [319] 10.1021/acsnano.0c05317

Theranostics aim to deliver point-of-care diagnosis and treatment with the same nanoformulation [313]. Theranostic agents can monitor the accumulation of nanomedicine compounds at the target site, visualize biodistribution, quantify triggered drug release, and assess therapeutic efficacy [314–316]. One of the most important aspects of theranostics is the capability to predict response in individual patients, thus paving the way for personalized medicine [317]. They may also offer a means of dealing with tumor heterogeneity since they can indicate the presence of a target and its exact location in the body [318]. The innovative concepts and strategies of theranostics have not yet been fully evaluated in clinical trials but there is a plethora of preclinical studies on the verge of clinical translation. Theranostic NPs were engineered by encapsulating the NIR-II nanofluorophore boron-dipyrromethene within amphipathic poly(styrene-co-chloromethylstyrene)-graft-poly-(ethylene glycol) nanocarriers functionalized with cell death-ligand 1 (PD-L1) monoclonal antibody (Fig. 8) [319]. Upon an 808 nm laser excitation, the targeted NPs produce an emission wavelength above 1200 nm to image a tumor to a normal tissue signal ratio (T/NT) at an approximate value of 14.1. These NPs exhibit high singlet oxygen quantum yield (ΦΔ = 73%), and an eliminating effect of primary cancers. The NPs also allow for profiling PD-L1 expression as well as accumulating in MC38 tumor and enabling molecular imaging in vivo. MC38 tumors in mice were eliminated by combining photodynamic therapy and immunotherapy within 30 days, with no tumor recurrence within a period of 40 days. In addition, the tumors do not grow in the rechallenged mice within 7 days of inoculation. These NPs showed durable immune memory effect against tumor rechallenging without toxic side effects to major organs. A proof-of-principle report showed agglomerated single-walled carbon nanotubes (SWCNTs) to be a potentially promising theranostic tool that allows for photoactivated destruction of cancer cells while keeping the local environment alive [320]. Absorptions of picosecond pulses of light by the SWCNTs creates photoacoustic induced cellular destruction without destroying the nearby environment allowing for continuous monitoring.

## Cancer radiation therapy

### Current approaches to radiotherapy

Approximately 60% of cancer patients receive radiation treatment during the course of disease, depending on cancer type, stage, and time of diagnosis [321]. Radiation therapy (RT) is highly utilized to combat cancer because of its effectiveness in inducing DNA damage and subsequent cellular death, particularly in rapidly dividing cancer cells [322]. Although highly effective, radiotherapy is still not localized enough to avoid harmful effects on other parts of the body. Combination chemoradiotherapy is standard of care for many types of cancer, but further increases likelihood of systemic toxicity, however certain technological advancements in the past few decades have led to significant improvements [323]. 3D conformal radiation treatments, such as stereotactic (body) radiotherapy, intensity-modulated RT and improved imaging systems (i.e., image-guided RT), coupled with superior understanding of tumor biology have increased cancer RT survival rates from 30 to 80% [3]. Lastly, some cancers are known to be resistant to radiotherapy thus utilizing nanomaterials to enhance and hone specificity can greatly reduce toxicity of treatment [324].

### Emerging nanotechnologies for RT in clinical applications

RT can benefit from nanotechnology enhancements since nanomaterials have specific properties conducive to atomic-level interactions with radiation and tumoral accumulation. High atomic number NPs have been shown to enhance Compton and photoelectric effects of conventional RT, and certain nanomaterials can utilize radiation-triggered drug release while others can serve as radiosensitizers [325, 326]. There are several clinical studies underway that utilize nanomaterials for enhancing RT further elucidated in this section (Table 4).

**Table 4:** Clinical trials utilizing nanomaterials to enhance radiation therapy

NCT Number	Title	Conditions	Interventions	Phases
NCT04862455	NBTXR3, radiation therapy, and Pembrolizumab for the treatment of recurrent or metastatic head and neck squamous cell cancer	Metastatic head and neck squamous cell carcinoma/recurrent head and neck squamous cell carcinoma	Other: Hafnium oxide-containing NPs NBTXR3/Radiation: hypofractionated radiation therapy/Biological: Pembrolizumab/Radiation: stereotactic body radiation therapy	Phase 2
NCT04789486	Nano-SMART: NPs With MR guided SBRT in NSCLC and pancreatic cancer	NSCLC/advanced pancreatic adenocarcinoma/unresectable pancreatic cancer/ductal adenocarcinoma of the pancreas	Drug: AGuIX/Radiation: radiotherapy	Phase 1/2
NCT04682847	Radiotherapy with iron oxide NPs (SPION) on MR-Linac for primary & metastatic hepatic cancers	Liver neoplasms/hepatic cirrhosis/hepatic carcinoma/liver cancer/liver metastases/liver carcinoma/hepatocellular carcinoma/hepatocellular cancer/hepatic atrophy	Drug: Ferumoxytol injection	
NCT04615013	NBTXR3, chemotherapy, and radiation therapy for the treatment of esophageal cancer	Cervical Esophagus Adenocarcinoma/Clinical Stage II esophageal adenocarcinoma AJCC v8/Clinical Stage IIA esophageal adenocarcinoma AJCC v8/Clinical Stage IIB esophageal adenocarcinoma AJCC v8/Clinical Stage III esophageal adenocarcinoma AJCC v8/Gastroesophageal Junction Adenocarcinoma/Pathologic Stage II esophageal adenocarcinoma AJCC v8/Pathologic Stage IIA esophageal adenocarcinoma AJCC v8/Pathologic Stage IIB esophageal adenocarcinoma AJCC v8/Pathologic Stage III esophageal adenocarcinoma AJCC v8/Pathologic Stage IIIA esophageal adenocarcinoma AJCC v8/Pathologic Stage IIIB esophageal adenocarcinoma AJCC v8/Thoracic Esophagus Adenocarcinoma	Drug: Capecitabine/Drug: Carboplatin/Drug: Docetaxel/Drug: Fluorouracil/Other: Hafnium oxide-containing NPs NBTXR3/Radiation: intensity-modulated radiation therapy/Drug: Leucovorin/Drug: Oxaliplatin/Drug: Paclitaxel	Phase 1
NCT04505267	NBTXR3 and radiation therapy for the treatment of inoperable recurrent NSCLC	Recurrent lung non-small cell carcinoma/Stage I lung cancer AJCC v8/Stage IA1 lung cancer AJCC v8/Stage IA2 lung cancer AJCC v8/Stage IA3 lung cancer AJCC v8/Stage IB lung cancer AJCC v8/Stage II lung cancer AJCC v8/Stage IIA lung cancer AJCC v8/Stage IIB lung cancer AJCC v8/Stage III lung cancer AJCC v8/Stage IIIA lung cancer AJCC v8/Stage IIIB lung cancer AJCC v8/Stage IIIC lung cancer AJCC v8/Unresectable lung non-small cell carcinoma	Other: Hafnium Oxide-containing NPs NBTXR3/Radiation: radiation therapy	Phase 1
NCT04484909	NBTXR3 activated by radiation therapy for the treatment of locally advanced or borderline-resectable pancreatic cancer	Borderline resectable pancreatic adenocarcinoma/locally advanced pancreatic ductal adenocarcinoma/resectable pancreatic ductal adenocarcinoma/stage III pancreatic cancer AJCC v8	Other: Hafnium Oxide-containing NPs NBTXR3/Radiation: radiation therapy	Phase 1
NCT04240639	An extension study MRI/US fusion imaging and biopsy in combination with nanoparticle directed focal therapy for ablation of prostate tissue	Neoplasms of the Prostate	Device: AuroShell particle infusion	Not Applicable
NCT03589339	NBTXR3 activated by radiotherapy for patients with advanced cancers treated with an anti-PD-1 therapy	Radiotherapy/immunotherapy/microsatellite instability-high solid malignant tumour/metastasis from malignant tumor of stomach (disorder)/squamous cell carcinoma of head and neck/metastasis from malignant tumor of cervix/metastatic squamous cell carcinoma/metastasis from malignant melanoma of skin (disorder)/merkel cell carcinoma (disorder)/metastasis from malignant tumor of lung/metastasis from malignant tumor of bladder (disorder)	Drug: NBTXR3	Phase 1
NCT02379845	NBTXR3 crystalline NPs and radiation therapy in treating randomized patients in two arms with soft tissue sarcoma of the extremity and trunk wall	Adult soft tissue sarcoma	Device: NBTXR3/Device: Radiation therapy	Phase 2/Phase 3
NCT01946867	NBTXR3 and radiation therapy in treating patients with locally advanced scc of the oral cavity or oropharynx	Head and neck cancer	Device: NBTXR3 activated by IMRT	Phase 1

AGuIX is a nanoparticle composed of polysiloxane-based inorganic matrix bound to chelating agent DOTA (1,4,7,10-tetra-azacyclododecane-1-glutaric anhydride-4,7,10-triacetic acid) covalently bound to the paramagnetic contrast enhancer Gd [327, 328]. Upon placement in a magnetic field, AGuIX produces a large magnetic moment and subsequently a large local magnetic field, which can enhance the relaxation rate of nearby protons, increasing MRI signal in tumor tissues where they have accumulated. The ultra-small NPs, less than 5 nm in diameter, allow for rapid renal clearance and reduced toxicity, and amplified radiation effects of AGuIX NPs were recently elucidated, attributed to the emission of low-energy photoelectrons and Auger electron interactions [329]. The preclinical study showed AGuIX NPs exacerbated radiation-induced DNA double-strand break damage and reduced DNA repair in the H1299 NSCLC cell line and is currently being tested in clinical trials (ClinicalTrials.gov Identifier: NCT04789486).

Certain NPs can combine radiation with localized PDT to induce tumor tissue destruction via ROS generation and radiosensitization, providing the benefit of physical ablation to deep tissue targets [330]. AuroLase therapy is a particle-directed photothermal therapy used with infrared-absorbing Auroshell NPs within tumor tissue to generate lethal doses of heat [331]. Auroshell particles consist of a gold metal shell surrounding a silica core, and a near-infrared (NIR)-tuned optical fiber can specifically deliver photonic laser energy which the NPs can absorb and create lethal photothermal lesions that are confined to the tumor tissue where Auroshells have accumulated [332]. In initial clinical studies, NP-mediated focal laser ablation was successful in 15/16 prostate cancer patients, and they are currently being investigated in an expanded clinical study (ClinicalTrials.gov Identifier: NCT04240639).

Normal X-ray radiation induces DNA damage through ROS generation after interaction with water molecules. NBTXR3 are hafnium oxide NPs engineered to increase energy deposit due to high electron density, thereby inducing greater oxidative stress within tumor cells and subsequent physical ablation [333]. Soft tissue sarcomas of limbs or trunk enable direct injection of NPs into the tumor, where the radiotherapy enhancement can be localized to cancerous tissue, but locally advanced soft tissue sarcomas (high risk that are typically unresectable) often requires pre-operative radiotherapy, making ideal cancer types for testing NBTXR3 [334]. In a recent phase 2/3 trial (ClinicalTrials.gov Identifier: NCT02379845), the rate of pathologic complete response (< 5% remaining viable tumor cells) was achieved in twice as many patients in test arm as in the control arm (16 vs 8%; P = 0.044), and the NPs were well tolerated. NBTXR3 is currently being evaluated in 8 clinical studies on various cancers (ClinicalTrials.gov Identifiers: NCT01946867, NCT04505267, NCT03589339, NCT04484909, NCT04615013, NCT04862455, NCT04834349, NCT04892173). Radiation-induced liver disease (RILD) or radiation hepatitis is a sub-acute form of liver injury due to radiation [335]. It is one of the most severe side effects of radiation which prevents radiation dose escalation and re-irradiation for hepatobiliary or upper gastrointestinal malignancies. Hepatic cirrhosis in patients with hepatocellular carcinomas (HCC), or chemotherapy-induced hepatic atrophy or hepatosteatosis in patients with liver metastases can be associated with high risk of RILD after stereotactic body radiotherapy (SBRT) [336, 337]. However, hepatotoxicity can be greatly reduced by switching to MRI-guided radiotherapy with SPION on 1.5 Tesla MR-Linac as opposed to nuclear medicine [338]. MRI-SPION radiotherapy is expected to facilitate detection and maximize avoidance of residual, functionally-active hepatic parenchyma from over-the-threshold irradiation, thus increasing safety of liver stereotactic body radiotherapy in patients with pre-existing liver conditions (ClinicalTrials.gov Identifier: NCT04682847).

### Nanomaterials to improve and augment RT

**Fig. 9:**
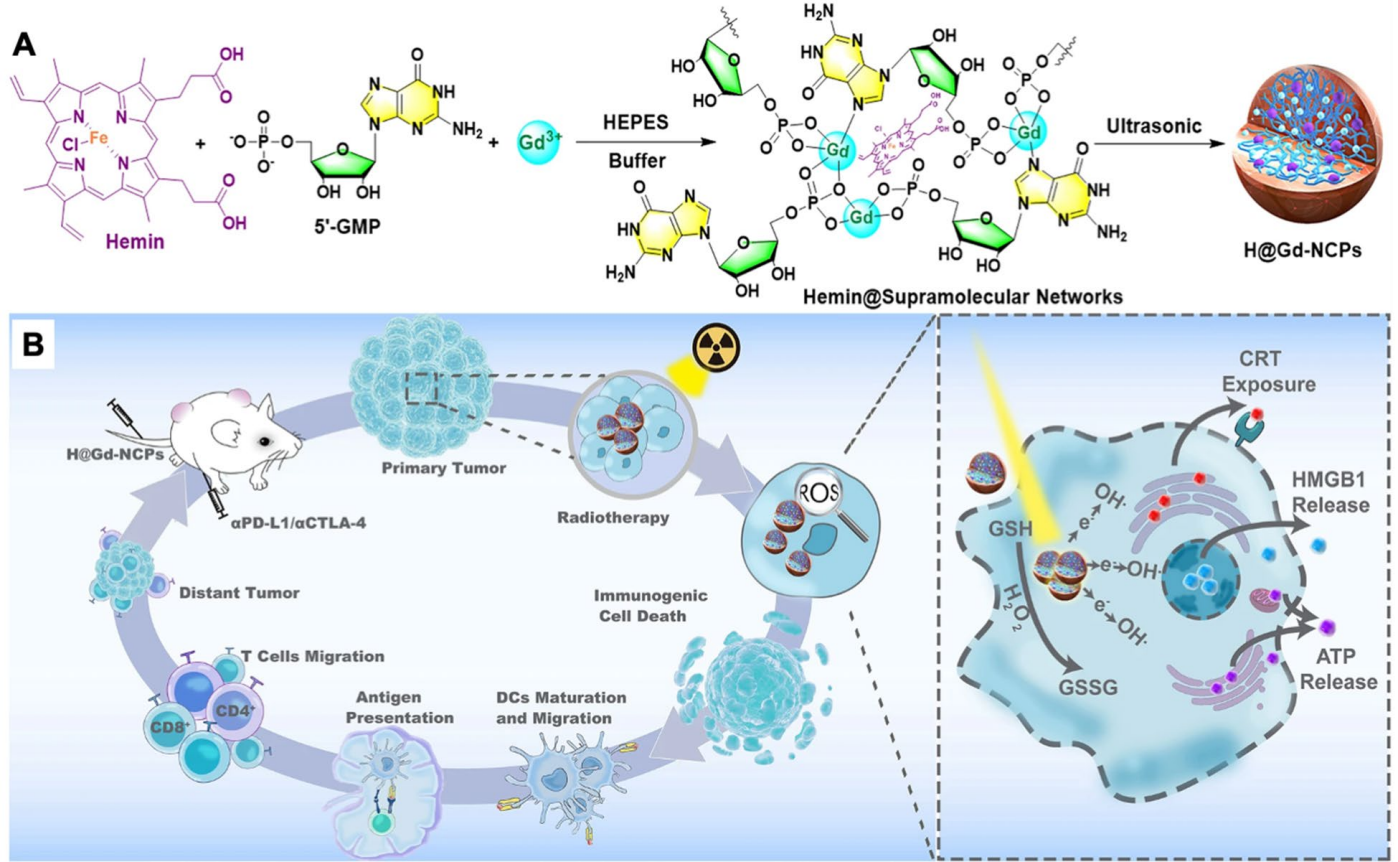
**A** Preparation of a novel radiosensitizer that incorporates nanoscale coordination polymers (NCPs) based on gadolinium (Gd^3+^) and 5′-Guanosine monophosphate (5′-GMP) via supramolecular self-assembly. **B** Mechanism of radiosensitization via amplification of radiotherapy-mediated oxidative stress. Dendritic cells (DCs), glutathione (GSH), oxidized glutathione (GSSG), hydroxyl radicals (•OH), calreticulin (CRT), high mobility group protein B1 (HMGB1), adenosine triphosphate (ATP). Reprinted with permission, [347] 10.1038/s41467-020-20243-8

Drug resistance and cancer heterogeneity can be addressed by physical destruction of tumor cells through RT, but there is room for improvement with respect to specificity and enhanced efficacy. Previous research has shown that radiotherapy can be utilized to activate the immune system by inducing immunogenic cell death (ICD), an immune response against the antigens of dead or dying tumor cells [339]. ICD-associated damage via ROS production could possibly promote the activation and migration of dendritic cells to prime T cells for systemic anti-tumor immune responses [340, 341]. However, radiation-stimulated immune responses have shown limited efficacy, particularly when tumors exhibit low X-ray absorption and energy deposition capacities [342, 343]. Disjoint oxygen supply and demand within tumors result in hypoxic areas with high levels of hydrogen peroxide, which induce adaptive antioxidant mechanisms [344, 345]. Subsequently, high concentrations of reducing substances, such as glutathione, quench •OH generated by RT, ultimately reducing its efficacy [346]. One solution to amplify RT mediated oxidative stress to induce ICD for antitumor immunity activation was developed via a novel radiosensitizer that incorporates nanoscale coordination polymers (NCPs) based on Gd^3+^ and 5′-Guanosine monophosphate (5′-GMP) via supramolecular self-assembly (Fig. 9) [347]. Hemin (PANHEMATIN®) with peroxidase-mimic catalytic activity was incorporated into the Gd^3+^/5′-GMP NCPs (Gd-NCPs) to form Hemin@ Gd^3+^/5′-GMP NCPs (H@Gd-NCPs). Furthermore, presence of metal element Gd, H@Gd-NCPs can act as an MRI contrast agent, adding to its utility for clinical use. The H@Gd-NCPs effectively enhance X-ray absorption and produce more ROS, especially hydroxyl radicals within tumor tissues. The encapsulated hemin can enhance peroxidase-like properties to utilize overexpressed hydrogen peroxide in TME to deplete GSH. Combination of ROS enhancement and GSH depletion amplifies irradiation-mediated oxidative stress and induce ICD. The antitumor immunity activated by H@Gd-NCPs can further be strengthened by immune checkpoint blockade therapy against primary, distant, and metastatic tumors. Another approach to address hypoxic environmental impact on RT is to utilize nitric oxide (NO) prodrugs, shown to be efficient radiosensitizers as cell respiration inhibitors, and co-delivery with exogenous oxygen resources [348, 349]. Recent work has shown potential solution to this obstacle through a hybrid semiconducting organosilica-based O_2_ nanoeconomizer which in an acidic tumor environment releases NO and, via mild photothermal treatment, releases O_2_ resulting in enhanced efficacy of radiotherapy in vitro and in vivo [350]. A semiconducting polymer brush (SPB) framework has an electron donor and acceptor backbone, providing NIR II fluorescence, photoacoustic contrast, and photothermal conversion for theranostic application. It can be tuned for mild hyperthermia with tumor oxygenation improvement to boost RT, or higher temperature physical ablation. A hybridized fluorocarbon (FC) chain provides ease for oxygen loading and photothermally-controlled release, and in situ polymerization of PEG and alkyl chains improves biocompatibility and loading/retention of NO prodrugs. This novel nanoplatform (pHPFON-NO/O_2_) demonstrates tunable, pH-activated NO release and radiation-activated O_2_ delivery for enhanced radiosenstivity. An alternative to photon irradiation is the use of fast ion beams (proton therapy and hadron therapy [70–400 MeV amu^−1^]) to treat solid tumors [351]. Since ion irradiation has more specific tumor targeting, they are generally used for tumors in highly sensitive tissues such as eyes and brain, pediatric cancers, and/or radioresistant tumors [352]. However, one significant drawback remains as the damage sustained to healthy tissue in front of the tumor, so radiosensitizers can amplify the radiation effects within the tumor area while lowering dosage to the healthy tissue. [353] A Gd-chelated polysiloxane matrix based-nanoparticle was recently engineered to increase dose effect through generation of a high number of radicals via direct or indirect interaction of high-energy particles with Gd [354].The efficiency of AGuIX NPs to amplify the effects of medical protons was demonstrated using a 150 MeV proton beam under two irradiation conditions mimicking the entrance (0.44 keV µm^−1^) and the end (3.6 keV µm^−1^) of the proton track on plasmid pBR322, and is currently under clinical investigation in France [327].

Hyperthermia (localized heat to kill cells) is a promising method for elimination of cancerous tissue, and certain nanomaterials have been shown to enhance hyperthermal effects [355, 356]. Uniform and selective hyperthermia can be achieved using nanomaterials with a high-absorption cross-section, which can convert an external energy source into heat [357, 358]. Dynamic nanomaterials can achieve maximum therapeutic effects through multi-modal cancer treatments. Multi-modal treatments pose the possibility of eliminating cancer via physical activation such as photothermal, photodynamic, radiation, and magnetic. Specifically, gold and carbon nanomaterials have been extensively used to induce hyperthermal effects upon near-infrared light (NIR) irradiation [248, 359]. They can further be utilized for triggered drug release with therapeutics tethered to the surface or encapsulated within, and payload can release upon change in temperature or irradiation [360, 361]. This approach can incorporate a combinatorial approach by using therapeutic agents in conjunction with an external trigger to localize treatment and/or destroy cancerous cells with physical ablation [362]. Recently, activatable polymeric pro-nanoagonist (APNA) triggers tumor ablation by the photothermal effect and induces immunogenic cell death and is activated by second near-infrared (NIR-II) light which enables deep-tissue penetration and represents a strategy for future RT [363]. APNA is constructed from covalent conjugation of toll-like receptor type 7 and 8 agonist (Resiquimod: R848) onto a NIR-II semiconducting transducer through a labile thermo-responsive linker. Upon NIR-II photoirradiation, APNA mediates photothermal effect, triggers tumor ablation, immunogenic cell death, and initiates the cleavage of thermolabile linker to liberate caged agonist for in-situ immune activation in deep solid tumor (8 mm). Cancer stem cells are particularly concerning due to their resistance for anticancer drugs, thus alternative methods are necessary [364]. A novel approach to utilizing nanomaterials with radiation is photothermal control of heat-sensitive TRPV1 or TRPV2 ion channels to regulate cell stemness [365]. This recent study demonstrates NIR-photoactive nanocarbon complexes can stimulate TRPV2 overexpression in cancer cells, disrupting intracellular Ca^2+^ regulation, suppressing Wnt/β-catenin signaling, which resulted in the destruction of cancer cells and inhibition of stemness in both in vitro and in vivo models.

Although photothermal agents (PTAs) have shown promising results in clinical studies, rapid degradation of PTA limits the photothermal stability required for efficacious treatment yet those with high photothermal stability degrade slowly thus have greater safety concerns [366, 367]. Currently, there are few PTAs with high photothermal stability and rapid degradation. Recently, it was shown that the inherent Cu^2+^-capturing ability of black phosphorus (BP) can accelerate the degradation of BP, while also enhancing photothermal stability [368]. The incorporation of Cu^2+^ into BP@Cu nanostructures further enables chemodynamic therapy-enhanced PTT. Moreover, by employing ^64^Cu^2+^, PET imaging can be achieved for in vivo real-time and quantitative tracking.

## Perspectives and conclusion

**Fig. 10:**
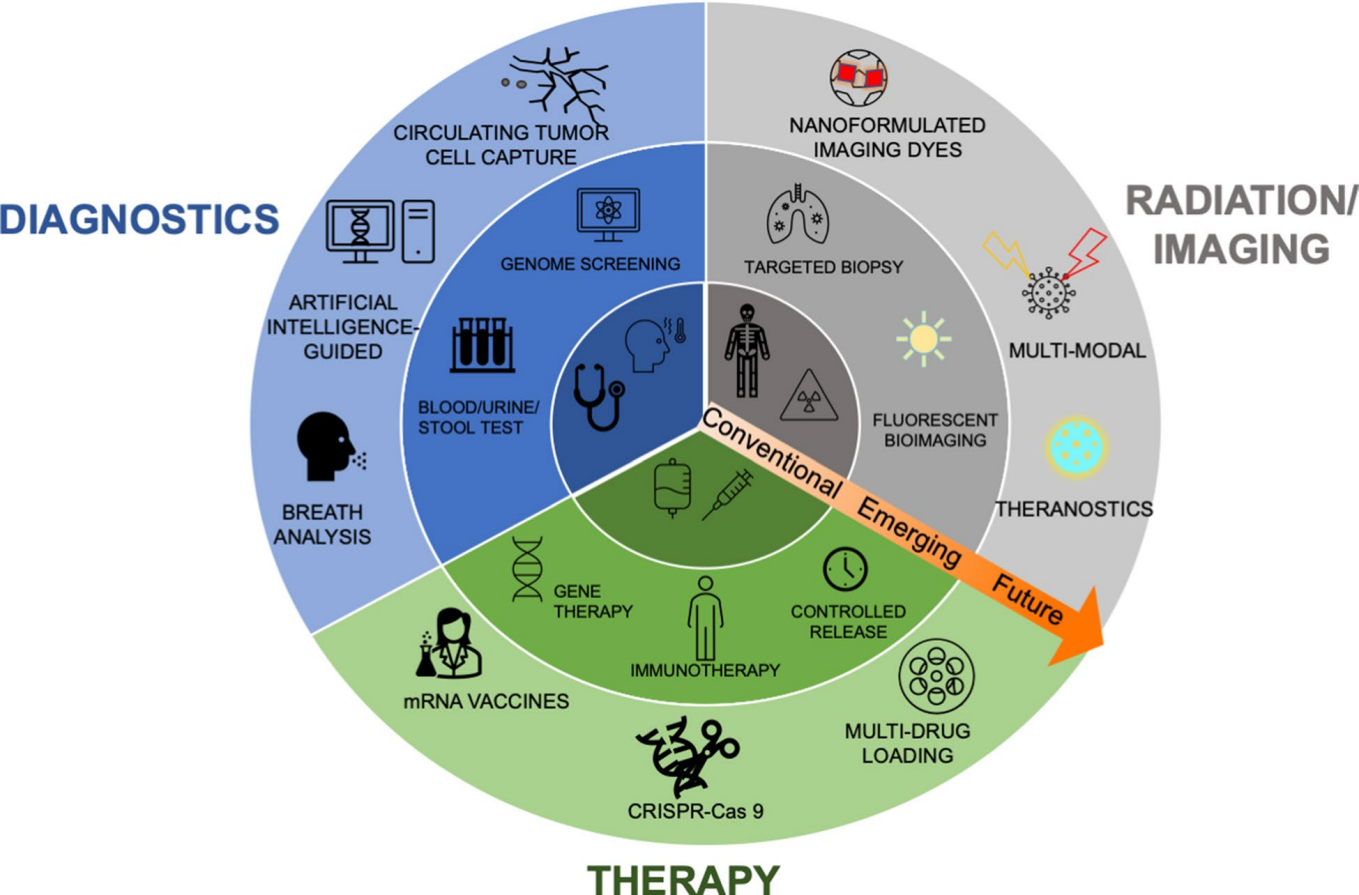
Conventional cancer therapies, diagnostics, radiation treatments, and imaging can be significantly improved through nanotechnological applications. Nanotechnologies are emerging in all fields at an increasing rate, and future applications hold great promise to significantly improve patient prognoses and quality of life

Nanomaterials are highly versatile, adaptable, and have many advantages that can improve cancer treatments and diagnostics (Fig. 10). However, factors such as production cost, scalability, safety, and complexity of nanoformulations must be considered and weighed against the potential benefits. As complexity of design and materials increases, so do costs, manufacturing criteria, and testing parameters [144]. Some nanomedicines may present a clear clinical benefit over conventional formulation, but if cost and production requirements are unattainable, clinical translation may never be realized. An increasingly important factor for commercial production is the environmental impact, not only of the nanomaterials themselves, but manufacturing by-products and energy costs [369]. In addition, there is a shadow of uncharted territory for FDA approval that many nanomedicines may face. The FDA has 3 product areas based on whether the product has a chemical mode of action (drug), a mechanical mode of action (device), or a biological source (biologic), and certain nanoformulations can span all 3 areas, categorizing them as a combination product. With rapidly advancing technologies for nanomedicine, there seems to be a need for more consistent and robust guidelines to evaluate clinical trials for nanomaterials. In 2006, the FDA Nanotechnology Task Force was created to address the regulatory deficiencies regarding nanoformulated medicines and devices, but determined that no new regulations were warranted [370]. The FDA published two documents regarding nanotechnology application and status, risk-based framework, specific requirements for conduct of nonclinical and clinical trials, manufacturing quality and controls, and environmental considerations [371, 372]. However, considering the pace and magnitude of nanotechnology research, a 15-year-old guidance is now exceedingly outdated. Although breakthrough status and subsequent accelerated approval can be achieved for certain drugs/devices/biologics, the gap in cohesive regulation for nanotechnology remains unclosed. Without comprehensive, updated evaluation and policy regarding nanotechnology in medicine and devices, the cost vs. benefit analysis will be unclear, and possibly a roadblock for critical research.

Cost vs. benefit analysis of nanomedicine poses many questions even without the issue of unclear regulatory guidelines. Nanomedicine can have much higher manufacturing costs than conventional drugs, depending upon formulation and complexity, however it is not as simple as comparing apples to apples [144]. Quality of life is typically only addressed for the duration of clinical trials; a recent study found that in only 5 of 149 studies (3.4%), quality of life was assessed until death, with only 1 out of 74 studies on metastatic or incurable cancers, and it is often not a co-primary endpoint [373]. Of course, quality of life may not be assigned a price, but certain quantifiable metrics may be applicable to evaluating the worth of nano-focused research and development. A 2011 survey found that one third of cancer survivors experienced limitations in their ability to perform usual daily activities outside of work, 25.1% felt cancer interfered with physical work tasks, and 24.7% overall felt less productive at work [374]. As previously noted, nanoformulations are often engineered to increase specificity, efficacy, and guard against drug resistance, thus patient quality of life is an important metric to evaluate for a prolonged period. There are many intricacies and considerations in determining the viability of drug products that go beyond merely cost of development vs. clinical outcome, and nanomedicine certainly adds to the equation.

Accessibility of information is at an apex, available at the touch of a button, which has greatly accelerated the advancement of scientific research. While extremely beneficial, it has also created a need for hierarchal and centralized organization as the scientific “toolbox” is flooded with data and new techniques. A search for the words “nano cancer” within only Nature publications for the year 2019 yielded 932 research articles, with Nature publications known to critically evaluate and publish highly impactful research. A recent study from MIT has constructed a machine learning framework to indicate “impactful” research that is overlooked by current metrics, opening the door to a possibility of machine-assisted direction of research [375]. While many in the scientific community were critical of the study and its implications, the idea of utilizing artificial intelligence to guide basic research has great potential. A multitude of parameters can be used to determine likelihood of clinical translation for nanotechnologies, as well as cross-reference with existing and emerging research to optimize formulation and strategy. Nanomedicine can greatly benefit from machine learning applications from analysis of patient tumor profiles and drug response to nanoparticle design, determining optimum material according to drug target, mechanistic attributes, and individualized prognoses [376, 377]. Machine learning was recently shown to estimate the cellular internalization of NPs based on their surface design, along with a machine learning-based model to sense breast cancer cells via internalization of eight differently functionalized carbon NPs (CNPs) [378]. The model accurately predicted the internalization of CNPs based on their structural features. NP cellular internalization were evaluated using different endocytic pathways, and artificial intelligence was then utilized to rank specific NP properties for optimum design. Furthermore, patient-specific cancer profiles were determined with machine learning techniques and cellular internalization profiles, demonstrating an efficient platform to render distinct fingerprints for individual cancer cell types. Neural networks are also demonstrating their use for diagnostics as well, from evaluating omics data to tumor imaging, and even optimizing radiotherapy [379–382].

The future of nanomedicine is certainly auspicious, with highly developed technologies improving treatments and diagnostics, and machine learning applications augmenting to save significant time and resources. There are multitudes of clinical and preclinical studies demonstrating the benefits of nanotechnology in cancer treatment, imaging, and diagnostics, but it is critical that these advances are clinically translatable. One key component in improving cancer patient outcome clearly lies in early detection methods. As previously discussed, early-stage cancers are generally much easier to treat, and early detection drastically improves 5-year survival rates and lowers patient cost. However, it is critical that diagnostic screenings are extremely accurate, otherwise misdiagnoses and overtreatments overshadow the benefits of early detection. Nanotechnology for cancer diagnostics, chemo- and radiotherapies stands to gain huge ground in the near future, creating a highly manageable cancer landscape for patients and oncologists. Although the dynamic nature of cancer refuses to yield, innovation continues to progress, and convergence of multiple technologies has promise to prevail.

## Data Availability

Not applicable.

## Abbreviations

PK/PD: Pharmacokinetics/pharmacodynamics
ADC: Antibody drug conjugate
AML: Acute myeloid leukemia
APC: Antigen presenting cells
APNA: Activatable polymeric pro-nanoagonist
ATO: Arsenic trioxide
BP: Black phosphorus
CAF: Cancer-associated fibroblast
CNP: Carbon nanoparticle
CPI: Checkpoint-inhibitor
CPT: Camptothecin
CT: Computed tomography
CTC: Circulating tumor cell
DCs: Dendritic cells
DOX: Doxorubicin
DRP: Drug Response Prediction
EGF: Epidermal growth factor
EGFR: Epidermal growth factor receptor
EMA: European Medicines Agency
EMT: Epithelial-mesenchymal transition
EpCAM: Epithelial cell adhesion molecule
EPR: Enhanced permeation and retention
EV: Extracellular vesicle
FAP: Fibroblast activation protein
FDA: U.S. Food and Drug Administration
HCC: Hepatocellular carcinoma
HLA: Human leukocyte antigen
HNCIB: High-throughput nano-biochip
HNSCC: Head and neck squamous cell carcinoma
HPV: Human papillomavirus
ICD: Immunogenic cell death
ICG: Indocyanine green
iNKT: Invariant natural killer T
LN: Lymph node
LNP: Lipid nanoparticle
mABs: Monoclonal antibodies
MRD: Minimal residual disease
MRI: Magnetic resonance imaging
MSN: Mesoporous silica NPs
NIR: Near-infrared
NIR-II: Second near-infrared
NO: Nitric oxide
NP: Nanoparticle
NY-ESO-1: New York esophageal squamous cell carcinoma 1
OMV: Outer membrane vesicle
PAA: Polyacrylic acid
PLGA: Poly(lacto-co-glycolic acid)
PLK1: Polo-like kinase 1
PTA: Photothermal agent
PTX: Paclitaxel
RILD: Radiation-induced liver disease
RNP: Ribonucleoprotein
RT: Radiation therapy
SBRT: Stereotactic body radiotherapy
sgRNA: Single-guide RNA
SIG: Signature
SLN: Sentinel lymph node
SMRT: Single molecule real time
SNALP: Stable nucleic acid lipid particles
SPION: Superparamagnetic iron oxide nanoparticle
SSR: Somatostatin receptors
SWCNT: Single-walled carbon nanotube
TAA: Tumor associated antigen
TCR: T cell receptors
TESLA: Target-Enabled in situ Ligand Assembly
TKI: Tyrosine kinase inhibitor
TME: Tumor microenvironment
TNBC: Triple negative breast cancer
Topo-1: Topoisomerase 1
TRAIL: Tumor necrosis factor-related apoptosis-inducing ligand
TSA: Tumor specific antigen
USPIO: Ultrasmall superparamagnetic iron oxide
VEGF: Vascular endothelial growth factor
VOC: Volatile organic compounds
